# Supplementary material for: PHD3 Regulates p53 Protein Stability by Hydroxylating Proline 359
Source: Cell Rep. 2018 Jul 31;24(5):1316–29. doi: 10.1016/j.celrep.2018.06.108 (PMC6088137; doi:10.1016/j.celrep.2018.06.108)
Supplement: Document S1. Supplemental Experimental Procedures and Figures S1–S6 [file mmc1.pdf]

Cell Reports, Volume *24*

Supplemental Information

PHD3 Regulates p53 Protein Stability

by Hydroxylating Proline 359

Javier Rodriguez, Ana Herrero, Shuijie Li, Nora Rauch, Andrea Quintanilla, Kieran Wynne, Aleksandar Krstic, Juan Carlos Acosta, Cormac Taylor, Susanne Schlisio, and Alex von Kriegsheim

Figure S1

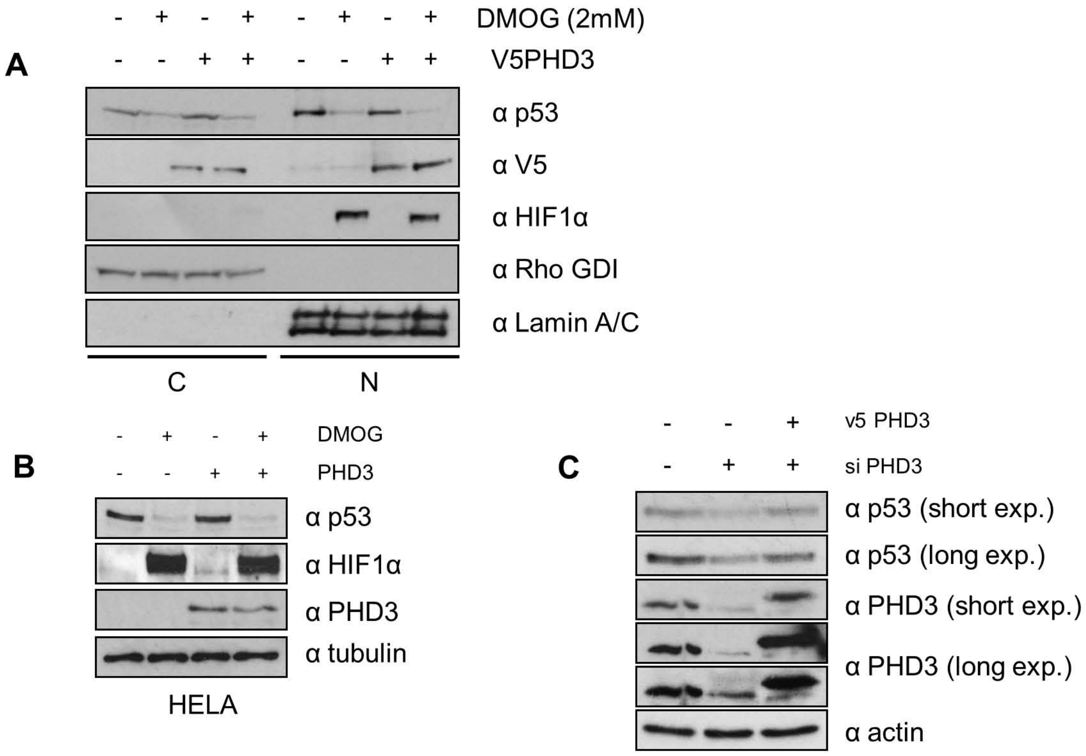

Figure S1 PHD3 regulates p53 protein stability. Related to Figure 2. (A) HeLa cells were transfected with an empty vector control and/or V5-tagged PHD3. 24 hours post-transfection the cells were treated or not with DMOG for 4 hours. Cells were lysed and the nuclear or cytoplasmic compartment were fractionated. Proteins were separated by PAGE, electroblotted and detected by the indicated antibodies. (B) HeLa cells were transfected with an empty vector control and/or V5-tagged PHD3. 24 hours post-transfection the cells were treated or not with DMOG for 4 hours. Cells were lysed and proteins were separated by PAGE, electroblotted and detected by the indicated antibodies.(C) HeLa cells were transfected with PHD3 specific siRNA. 24 hours post-transfection the cells were transfected with V5-PHD3 or not. Cells were lysed and proteins were separated by PAGE, electroblotted and detected by the indicated antibodies.

Figure S2

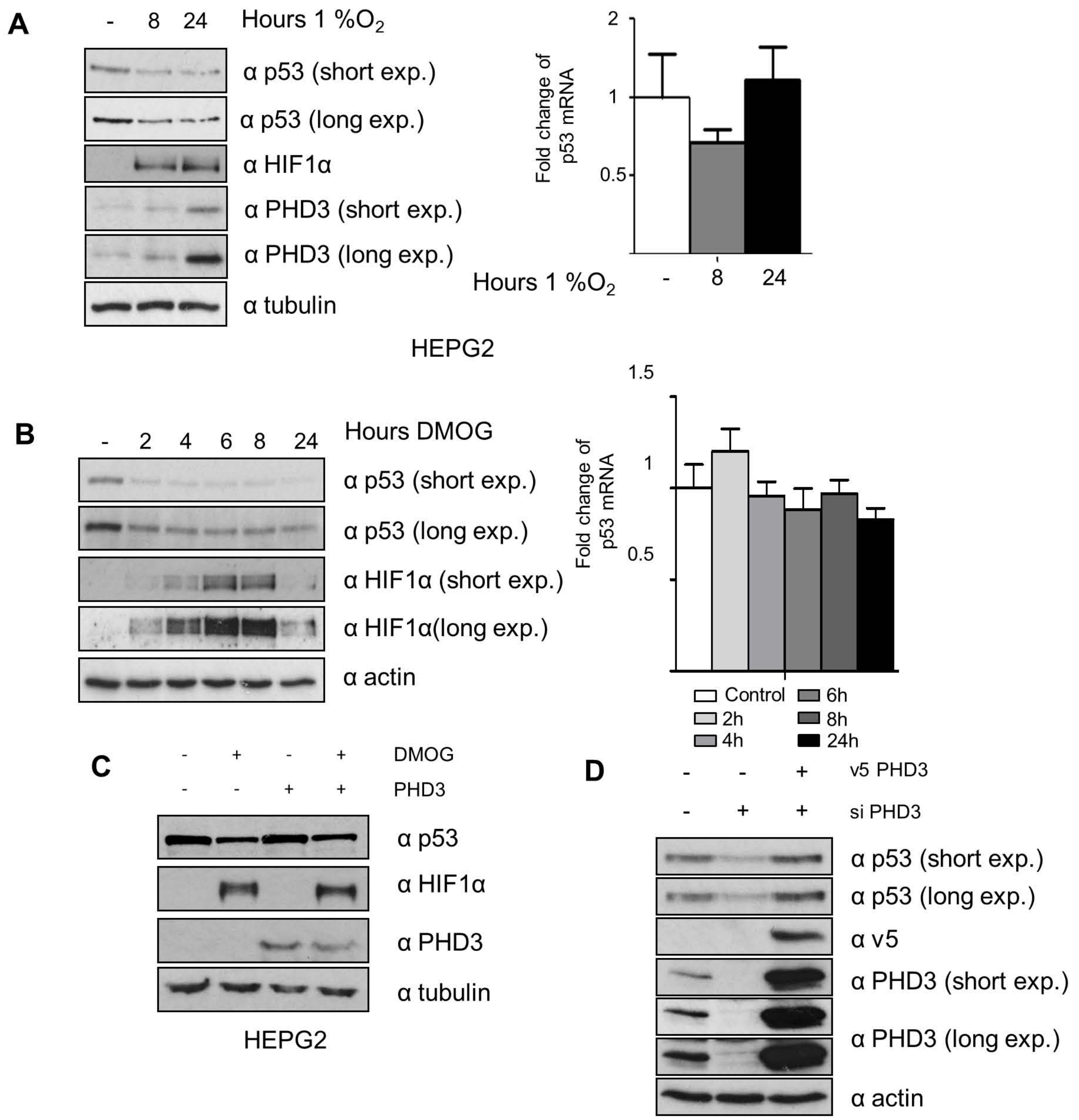

Figure S2 PHD3 regulates p53 protein stability and ubiquitination. Related to Figure 3. (A) HepG2 cells were cultured in 1% oxygen as indicated, lysed and proteins were separated by PAGE, electroblotted and detected by the indicated antibodies. In parallel, mRNA was extracted and quantified by RT-PCR (second panel) (B) HepG2 cells were treated with DMOG as indicated lysed and proteins were separated by PAGE, electroblotted and detected by the indicated antibodies. In parallel, mRNA was extracted and quantified by RT-PCR (second panel). (C) HepG2 cells were transfected with an empty vector control and/or V5-tagged PHD3. 24 hours post-transfection the cells were treated or not with DMOG for 4 hours. Cells were lysed and proteins were separated by PAGE, electroblotted and detected by the indicated antibodies. .(D) HepG2 cells were transfected with PHD3 specific siRNA. 24 hours post-transfection the cells were transfected with V5-PHD3 or not. Cells were lysed and proteins were separated by PAGE, electroblotted and detected by the indicated antibodies.

Figure S3

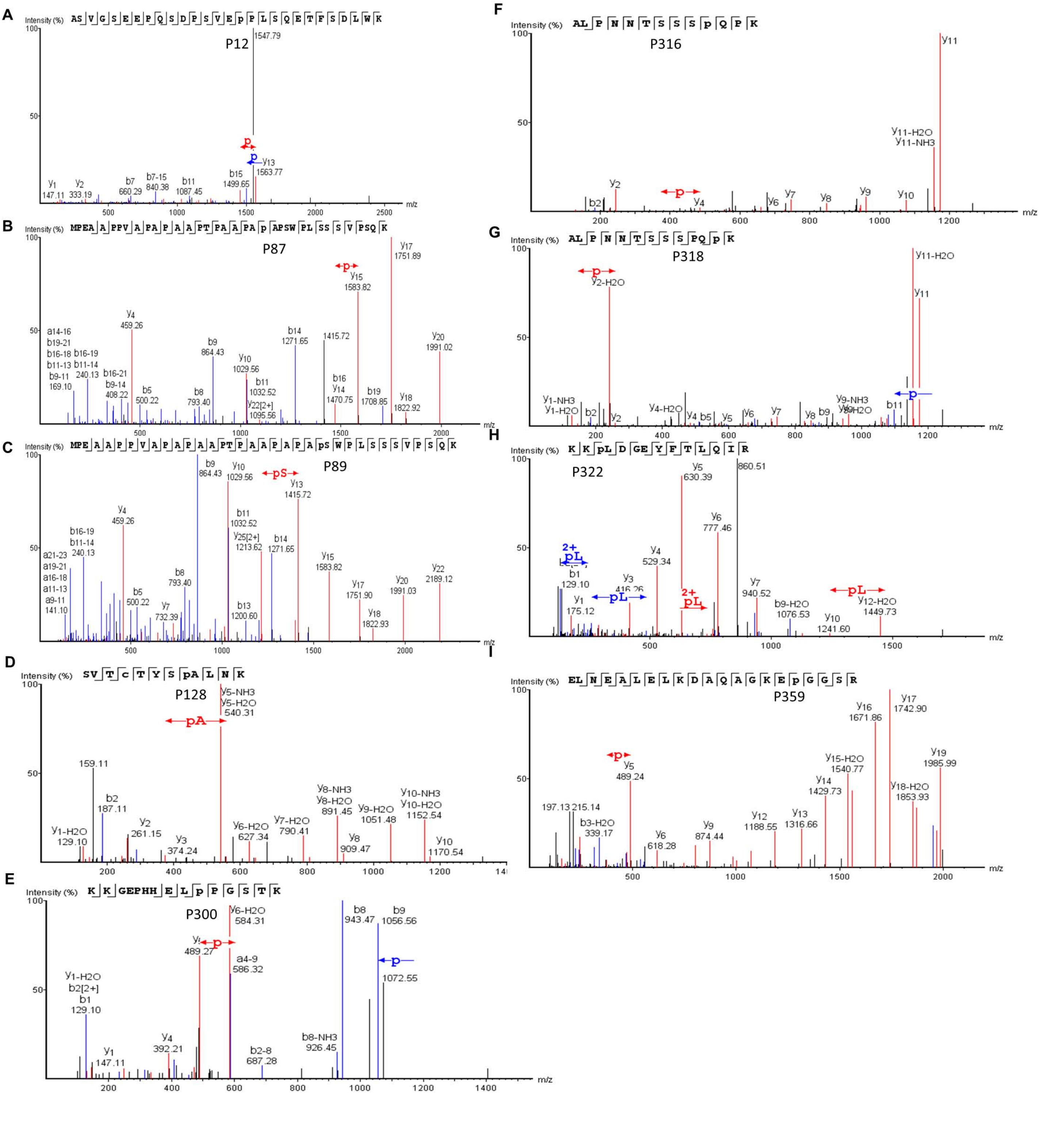

A
Intensity (%) ASVGSEEPQSDPSVEpPLSQETFSDLWK
P12
1547.79
p
p
y13 1563.77
y1 147.11
y2 333.19
b7 660.29
b7-15 840.38
b11 1087.45
b15 1499.65
m/z
B
Intensity (%) MPEAAPPVAPAPAAPTPAAPApAPSWPLSSSVPSQK
P87
p
y15 1583.82
y17 1751.89
y4 459.26
a14-16 b19-21 b16-18 b16-19 b11-13 b11-14 240.13
b9-11 169.10
b16-21 b9-14 408.22
b5 500.22
b8 793.40
b9 864.43
y10 1029.56
b11 1032.52
y22[2+] 1095.56
b14 1271.65
1415.72
b16 y14 1470.75
b19 1708.85
y18 1822.92
y20 1991.02
m/z
C
Intensity (%) MPEAAPPVAPAPAAPTPAAPAPApSWPLSSSVPSQK
P89
pS
b9 864.43
y10 1029.56
y13 1415.72
y4 459.26
b16-19 b11-14 240.13
a21-23 a19-21 a16-18 a11-13 a9-11 141.10
b11 1032.52
y25[2+] 1213.62
b14 1271.65
b8 793.40
b5 500.22
y7 732.39
b13 1200.60
y15 1583.82
y17 1751.90
y18 1822.93
y20 1991.03
y22 2189.12
m/z
D
Intensity (%) SVTCTYSpALNK
P128
pA
y5-NH3 y5-H2O 540.31
159.11
b2 187.11
y2 261.15
y1-H2O 129.10
y3 374.24
y6-H2O 627.34
y7-H2O 790.41
y8-NH3 y8-H2O 891.45
y8 909.47
y9-H2O 1051.48
y10-NH3 y10-H2O 1152.54
y10 1170.54
E
Intensity (%) KKGEPHHELpPGSTK
P300
y6-H2O 584.31
p
489.27
a4-9 586.32
b8 943.47
b9 1056.56
p
1072.55
y1-H2O b2[2+] b1 129.10
y1 147.11
y4 392.21
b2-8 687.28
b8-NH3 926.45
m/z
F
Intensity (%) ALPNNTSSSpQPK
P316
y11
y11-H2O y11-NH3
y2
p
b2
y4
y6
y7
y8
y9
y10
m/z
G
Intensity (%) ALPNNTSSSPQpK
P318
p
y2-H2O
y11-H2O
y11
y1-NH3 y1-H2O
b2
y2
y4-H2O
y4
b5
y5
y6
y7
y8
b9
y9-NH3 y9-H2O
p
b11
m/z
H
Intensity (%) KKpLDGEYFTLQIR
P322
y5 630.39
860.51
y6 777.46
2+ pL
b1 129.10
y4 529.34
y3 416.26
pL
2+ pL
y1 175.12
y7 940.52
b9-H2O 1076.53
y10 1241.60
pL
y12-H2O 1449.73
m/z
I
Intensity (%) ELNEALELKDAQAGKEpGGSR
P359
y16 1671.86
y17 1742.90
p
y5 489.24
y19 1985.99
y15-H2O 1540.77
y14 1429.73
y18-H2O 1853.93
197.13 215.14
b3-H2O 339.17
y6 618.28
y9 874.44
y12 1188.55
y13 1316.66
m/z

Figure S3. Fragmentation spectra of proline hydroxylated peptides detected in following an *in vitro* hydroxylation assay with GSTp53 as substrate. Related to Figure 4.

(A) ASVGSEEPQSDPSVEP(ox)PLSQETFSDLWF,

(B) MPEAAPPVAPAPAAPTPAAPAP(ox)APSWPLSSSVPSQK,

(C) MPEAAPPVAPAPAAPTPAAPAP(ox)AP(ox)SWPLSSSVPSQK

(D) SVTCTYSP(ox)ALNK

(E) KKGEPHHELP(ox)PGSTK

(F) ALPNNTSSSP(ox)QPK

(G) ALPNNTSSSPQP(ox)K

(H) KKP(ox)LDGEYFTLQIR

(I)ELNEALELKDAQAGKEP(ox)GGSR

Figure S4

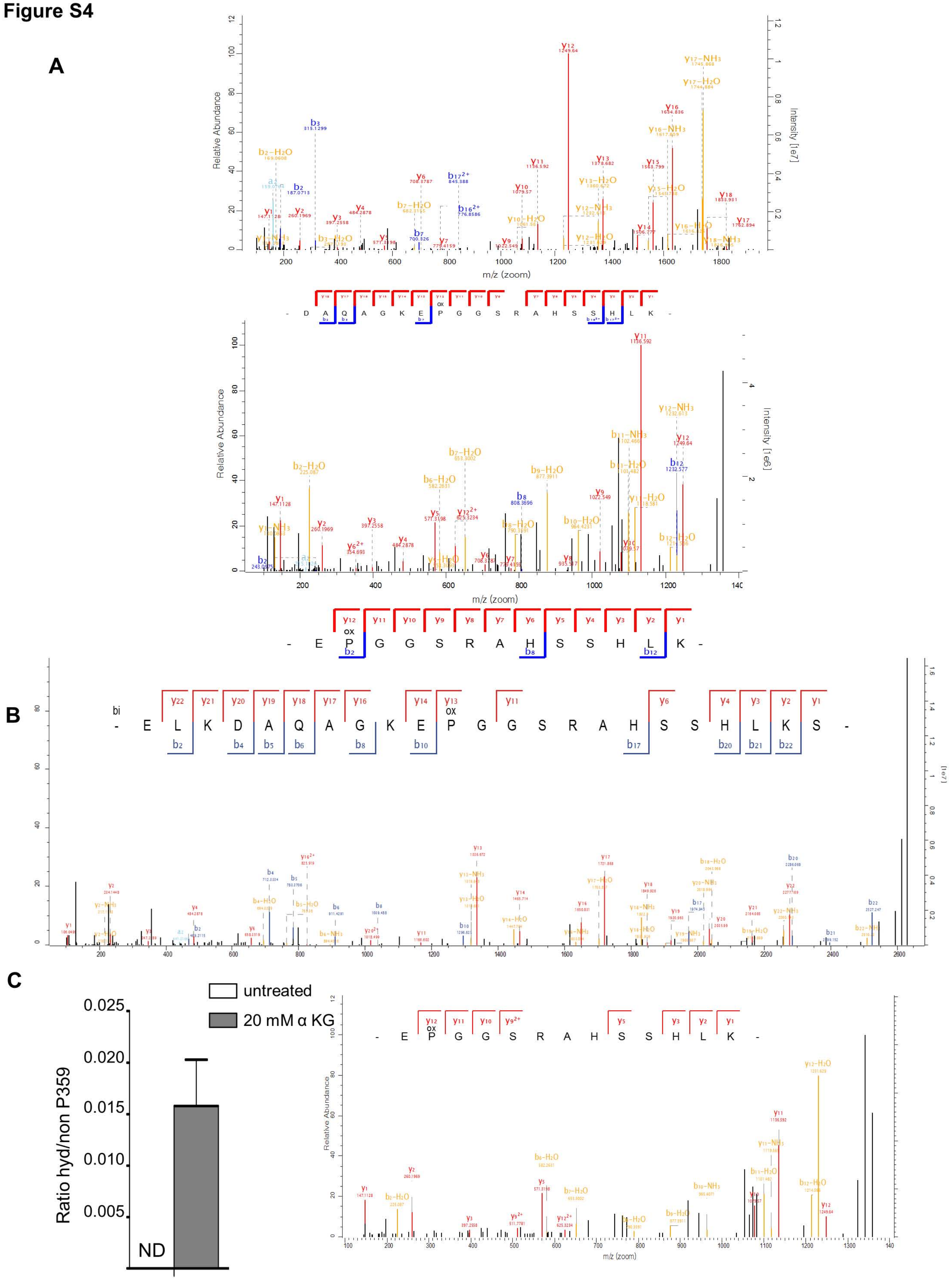
A
Relative Abundance
Intensity [1e7]
m/z (zoom)
- D A Q A G K E P G G S R A H S S H L K -
ox
Relative Abundance
Intensity [1e6]
m/z (zoom)
- E P G G S R A H S S H L K -
ox
B
bi
- E L K D A Q A G K E P G G S R A H S S H L K S -
ox
C
untreated
20 mM α KG
Ratio hyd/non P359
ND
- E P G G S R A H S S H L K -
ox
Relative Abundance
m/z (zoom)

Figure S4. Representative fragmentation spectra of the oxidation of p53 at the P359. Related to Figure 4.

(A) HCD fragmentation spectra of the peptide EP(ox)GGSRAHSSHLK and DAQAGKEP(ox)GGSRAHSSHdetected in a FLAG p53. (B) Fragmentation spectra of the biotinylated peptide ELKDAQAGKEP(ox)GGSRAHSSHLK. (C) Ratio of hydroxylated/non-hydroxylated peptide following an in vitro hydroxylation reaction with 20 mM alpha-ketoglutarate and the untreated peptide. Fragmentation spectra of EP(ox)GGSRAHSSHLK derived from the trypsin digested, biotinylated peptide ELKDAQAGKEPGGSRAHSSHLKS hydroxylated in vitro.

Figure S5

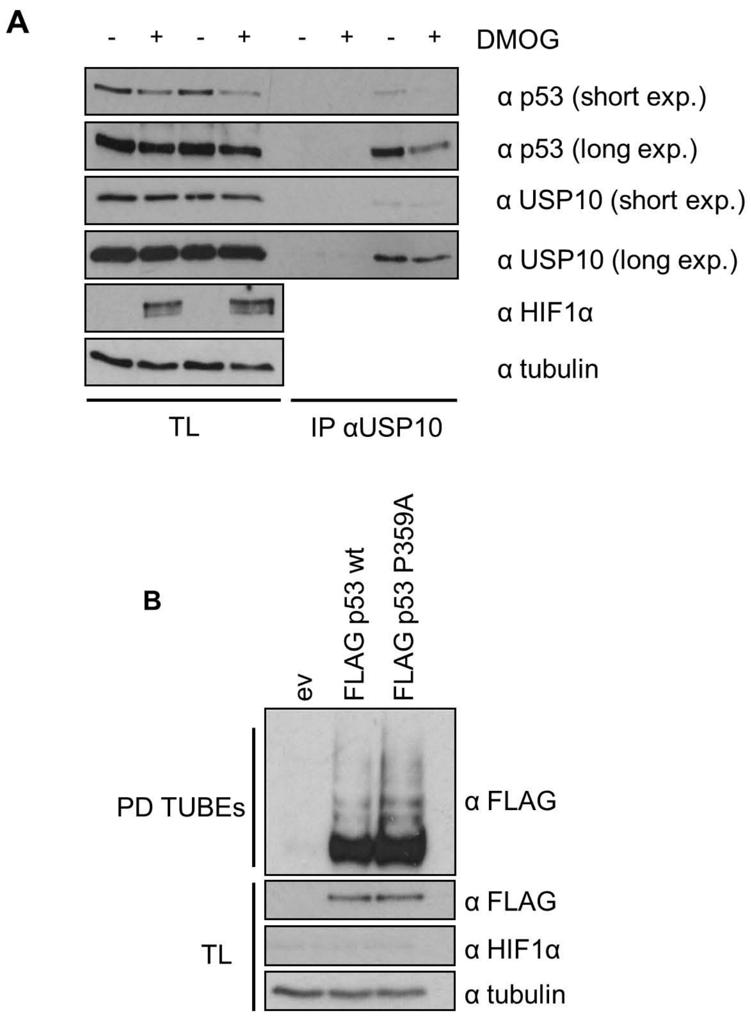

Figure S5. P53 and USP10 interact endogenously. Related to Figure 5.
(A) HepG2 cells were treated with or without DMOG for 4 hours. The cells were lysed and endogenous USP10 was immunoprecipitated. Total lysates and the corresponding immunoprecipitates were probed for the indicated proteins.
(B) HepG2 cells were transfected with Flag-p53 wt and Flag-p53 P359A. Cells were lysed and ubiquitinated proteins were precipitated with TUBE-agarose (PD). Proteins were separated by PAGE and electroblotted. Ubiquitinated Flag-p53 was detected by an anti-Flag antibody (PD) and changes in expression in total lysate were blotted separately (TL).

Figure S6

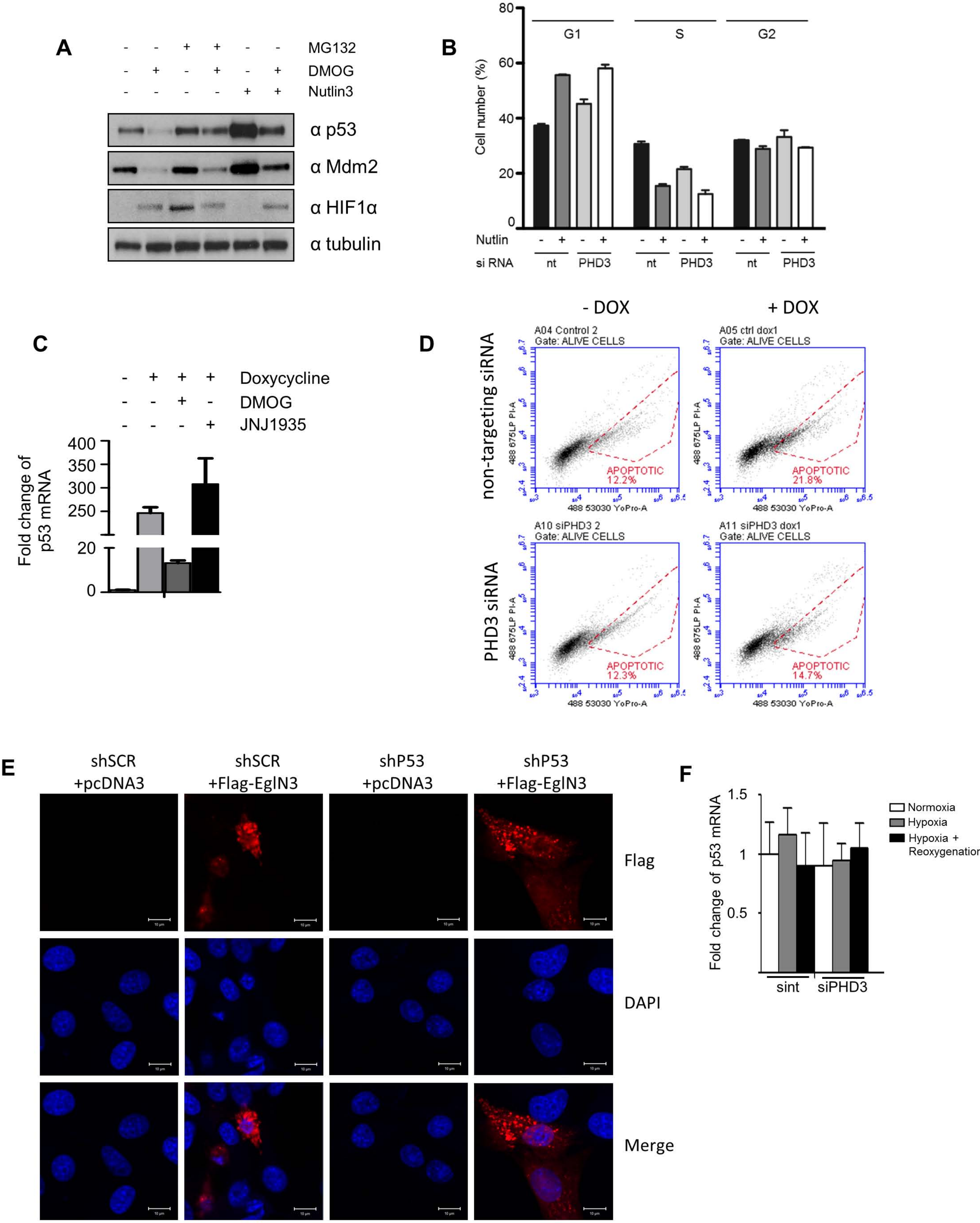
A
MG132
DMOG
Nutlin3
α p53
α Mdm2
α HIF1α
α tubulin
B
G1
S
G2
Cell number (%)
Nutlin
si RNA
nt
PHD3
C
Doxycycline
DMOG
JNJ1935
Fold change of p53 mRNA
D
- DOX
+ DOX
non-targeting siRNA
PHD3 siRNA
A04 Control 2
A05 ctrl dox1
A10 siPHD3 2
A11 siPHD3 dox1
Gate: ALIVE CELLS
APOPTOTIC
12.2%
21.8%
12.3%
14.7%
488 675LP PI-A
488 53030 YoPro-A
E
shSCR +pcDNA3
shSCR +Flag-EglN3
shP53 +pcDNA3
shP53 +Flag-EglN3
Flag
DAPI
Merge
F
Normoxia
Hypoxia
Hypoxia + Reoxygenation
Fold change of p53 mRNA
sint
siPHD3

Figure S6. PHD3 regulates p53 levels and downstream signalling. Related to Figure 6.
(A) HepG2 cells were treated with DMSO, DMOG or MG132 for four hours. Total lysates were analysed by Western blot with the indicated antibodies. (B) HepG2 cells were transfected with non-target or PHD3 siRNA. After 48 hours cells were treated for 4 hours with Nutlin3. Bar graphs represent the % of cells in G1, G2 and S. Error bars are SEM n=3. (C) p53-null Saos-2 were treated with doxycycline (1.25 µg/ml) for 24 hours. After this time these cells were treated with the combination of hydroxylases inhibitors that is showed at the panel. Cells were harvested for quantitative RT-PCR assays. The expression level of p53 was normalized to endogenous actin mRNA levels. The values plotted are means, SD of N=3 independent experiments for each condition. (D) p53-null Saos-2 were transfected with either siNT or siPHD3 for 24 h prior to 32 h treatment with Doxycycline (1.25 µg/ml). Representative graphs showing gating for apoptotic cells measured by YoPro/propidum iodide co-staining to detect apoptotic cells. (E) U87 cells stably infected with scrambled or p53-specific shRNA constructs were transiently transduced with plasmids expressing exogenous Flag-PHD3 expression or a control vector. Cells were stained with anti-Flag antibody or DAPI for nuclei. (F) HepG2 cells were transfected with non-target or PHD3 siRNA. After 24 hours cells were cultured in 1% oxygen (HY) or in normoxia (NO). Half of the hypoxic cells were taken out of hypoxia and culture for 60 minutes in normoxia (RO). The expression level of p53 was normalized to endogenous actin mRNA levels. The values plotted are means SD of N=3 independent

Supplemental Experimental Procedures

Cell culture: HEK293T, U87 and p53-inducible human tumour Saos-2 cells were cultured in Dulbecco's modified Eagle medium (DMEM) supplemented with 2 mM glutamine (Invitrogen) and 10% foetal calf serum (Invitrogen). HepG2 were cultured in the same conditions, but using the DMEM low glucose (1 g/L). Plasmids and siRNA oligonucleotides were transfected with Lipofectamine 2000 (Invitrogen) according to the vendor's instructions. P53-inducible Saos-2 cells were a kind gift from Dr Bob Ludwig (Prof. Vousden's group).

Materials: All antibodies were from commercial sources: anti-FLAG M2 peroxidase was obtained from Sigma Aldrich (F1804), anti-HIF1α was from BD Biosciences (610958), anti-p53, anti-tubulin, anti-GST, anti-HA, anti-His, anti-GST and anti-p21 were purchased from Santa Cruz(sc-126/sc-8035/sc-138/sc-7392/sc-803/sc-138/sc-6246, 1:1,000 dilution), anti-PHD3 was from Novus Biologicals (NB100-139), anti-USP10, anti-cleaved caspase 3 (Asp175) and anti-p53 was purchased from Cell Signalling Technologies (5553, 9664, 9282), and anti-V5 was obtained from Invitrogen (R96025). DMOG was obtained from Cayman Chemical (71210), MG132 was purchased from Sigma (M7449), the PHD-specific inhibitor JNJ-42041935 was purchased from Merck Millipore (400093), Nutlin3 was from Sigma (N6287), and cycloheximide was from Sigma (C4859/M7449).

Plasmids and siRNAs: HA-PHD3 and HA-PHD3 H196A were purchased from Addgene (18960/22717), and PHD3 H135A/D137A was a gift from Prof. Semenza Flag-USP7 and Flag-USP10 were from Addgene (16655/22543). FLAG-His-p53 was a gift from Cynthia Coffill. Flag–His-p53-P359A mutant was made using the QuikChange kit (Stratagene) using following oligos
CTAGGTATATGGACTTAAAAGCCTTGGGTTGTGGAGGCAATG (forward)
CATTGCCTCCACAACCCAAGGCTTTTAAGTCCATATACCTAG (reverse). The non-targeting siRNA (siNT), the siRNA targeting PHD3 (siPHD3) and the siRNA targeting USP10 were purchased from Dharmacon (ONTARGETplusSMARTpool).

Cell lysis and immunoprecipitation: Cells were lysed in ice-cold lysis buffer (1% Triton-x100, 20 mM Tris-HCl (pH 7.5), 150 mM NaCl), supplemented with protease (5 μg/ml leupeptin, 2,2 μg/ml aprotinin) and phosphatase inhibitors (20 mM β-glycerophosphate). Lysates were cleared of debris by centrifugation at 20,000 x g for 10 min in a benchtop centrifuge (4ºC). For immunoprecipitation anti-Flag-M2 beads (Sigma Aldrich) or anti-V5 beads (Sigma-Aldrich) were added to the cleared lysates and incubated at 4ºC under end-to-end rotation for 2 hours. Beads were washed three times with PBS 1X-1% NP40 and boiled off in Laemmli buffer.

Immunoblotting: Total lysates and affinity precipitates were fractionated by SDS-PAGE and transferred onto nitrocellulose filters. Immuno-complexes were visualized by enhanced chemiluminescence detection (GE Healthcare) with horseradish peroxidase–conjugated secondary antibodies (Bio-Rad Laboratories). Experiments were repeated at least 3 times.

Endogenous co-immunoprecipitation assays: Cells were washed in ice-cold PBS and lysed in 1% Triton-x100, 20 mM Tris-HCl (pH 7.5), 150 mM NaCl, supplemented with protease (5 μg/ml leupeptin, 2,2 μg/ml aprotinin) and phosphatase inhibitors (20 mM β-glycerophosphate). Lysates were cleared by centrifugation, 0.1 vol was separated to be loaded as the total lysate, and the rest were incubated with antibody rocking at 4°C for 1 h (a pre-immune antibody was used as negative control). Protein G–Sepharose was added and agitated at 4°C for 2 h. Beads were collected and washed three times with NP-40/PBS. Pellets were resuspended in 5× Laemmli buffer, boiled for 5 min, and fractionated by SDS-PAGE.

Mass spectrometry: HEK293T cells were transfected with either empty vector, a V5-tagged hydroxylases or Flag-tagged p53 and treated 24 hours post-transfection with either 2 mM DMOG or DMSO for 4 hours. The cells were lysed and we immunoprecipitated the protein with anti-V5 or anti FLAG agarose for 2 hour. The samples were digested with trypsin or Lys-C and processed as previously described (Turriziani et al., 2014). Desalted peptides were analysed on a Fusion Lumos mass spectrometer (Thermo, Germany).

In vitro hydroxylation assays:

Peptide hydroxylation: N-terminally biotinylated synthetic peptide ELKDAQAGKEPGGSRAHSSHLKS (Peptides & elephants, Germany) were incubated with streptavidin beads for 1 hour, after this incubation were washed 3 times with PBS. Finally were incubated with lysates derived from HEK293T cells transiently transfected with PHD3 wt or

PHD3 H196A as previously described. After the reaction was completed the peptides were eluted with biotin, desalted and analysed by LC-MS/MS.

p53 in vitro hydroxylation: The immobilized GST-p53 protein was incubated at 30°C for 1 h with HEK293T cell lysates supplemented with 100 µM FeCl2, 5 mM ascorbate and 1 mM α-ketoglutarate. After this incubation the beads were washed, digested with trypsin as described (Turriziani et al., 2014) and analysed by LC-MS/MS.

***In Vitro* Binding Assays:** GST fusion proteins were expressed in E. coli and purified. HEK293T cells transiently were transfected with V5-PHD3 and treated with 2 Mm DMOG or DMSO. Co-IP, GST pull-down, and immunoblot assays were performed as described (Rodriguez J et al., 2010).

Peptide pulldown assay: Peptides were purified with Streptavidin sepharose (Amersham Biosc.), and incubated with lysates of HEK293T cells that were transiently transfected with the indicated plasmids. GST pull-down, and immunoblot assays were performed as described (Rodriguez J et al., 2010).

Cell cycle analysis by PI staining: HepG2 cells were transfected with the specific siRNA for PDH3 (60pmol). After 48 hours, cells were treated with Nutlin-3 (10µM) for 5 hours prior to collection. After trypsinization (0.05% Trypsin-EDTA) and collection in growth medium (5ml), cells were washed once with ice-cold PBS, and finally re-suspended in 100 µl PBS. Addition of 900 µl of ice-cold ethanol (70%) was performed for cellular fixation with overnight incubation at 4 C. Prior Fluorescence-activated cell sorter (FACS) analysis, cells were re-suspended in 300 µl PBS containing Propidium Iodide (10 µg/ml) and RNAse A (100 µg/ml) and incubated in dark at room temperature for 30 minutes. Each sample was analysed, in duplicates, with BD Accuri C6, using FL-3 for PI (ex 488; em 670LP nm). Cells were gated by forward and side scatter in order to remove debris and doublets, resulting in the final "singlets in scatter" gating, and the DNA content per cell was measured using the BDAcuri software.

Apoptosis analysis by YO-PRO1/PI staining: p53-inducible Saos cells were transfected with siRNA for specific knockdown of PHD3 (60pmol) and treated with doxycycline (1.25 µg/ml) for 36 hours to induce p53 expression. Apoptosis levels were measured by Yo-Pro-1 uptake, which only permeates the membranes of apoptotic cells, and fluorescently labels the nucleus. Cells were collected by trypsinization (0.05% Trypsin-EDTA) in growth medium (5ml). Cells were washed with ice-cold PBS and incubated in PBS containing Yo-Pro-1 (Thermo Fisher Scientific) at 100 mM concentration. After 5 minutes Propidium Iodide at 10 µg/ml was added for another 5 minutes. Apoptotic levels were measured by assessing Fluorescence-activated cell sorter (FACS) in BDAccuri cytometer. Cells were gated by forward and side scatter for removing debris and doublets, and apoptotic cells were determined by Yo-Pro at FL-1 (ex 488; em 530±30 nm) and PI at FL-3 (ex 488; em 675LP nm) fluorescence.

rtPCR: Total RNA was isolated using RNAeasy Mini Kit (Quiagen) following the manufacturer's instructions, and the concentration and purity of the RNA samples were determined using the Nanodrop Spectrophotometer 2000c. The cDNAs were synthesized by qScript cDNA Supermix (Quanta Biosciences) according to the protocol, followed by a quantitative polymerase chain reaction performed on the StepOnePlus Real-Time PCR System (Applied Biosystems). The thermal cycling conditions were composed of an initial de-naturalisation at 95°C for 10 min, followed by 40 cycles at 95°C for 15 sec, 60°C for 30 sec and 72°C for 30 sec. The amplifications were done using the SYBR Select Master Mix (Applied Biosystems).

The expression of TP53 was normalized against beta-actin with the following primers (TP53 Fw 5'-CCGCAGTCAGATCCTAGCG-3'; TP53 Rv 5'- AATCATCCATTGCTTGGGACG-3'; beta-actin Fw 5'-CATGTACGTTGCTATCCAGGC-3'; beta-actin Rv 5'-CTCCTTAATGTCACGCACGAT-3').

Apoptosis Assay: Apoptosis was quantified using Hoechst staining to visualise apoptotic nuclei. Immunofluorescence staining for Flag allowed for visualization and quantification of Flag-positive cells via microscopy. 72 hours after transient transfection with Flag-PHD3 or Flag empty vector, U87 cells were incubated with Hoechst for 30 mins in incubator (37°C, 5% CO2). Cells were then fixed by 4% paraformaldehyde (PFA) for 15 mins and permeabilized by 0.1% Triton (0,1%) for 15 mins. Anti-Flag (mouse, 1:1000) was added to cells for overnight at 4°C. Secondary anti-mouse (Invitrogen, 1:1000) was added for 1 hour at room temperature. Approximately 200 Flag-positive cells were counted.

Crystal violet staining: U87 stable cell lines were transiently infected with adenovirus for PHD3 overexpression. Cells were then fixed and stained by crystal violet solution (0.1% crystal violet, 20% methanol, 80% dH2O) for 30mins at room temperature and washed 4 times with PBS.

Nuclear Cytoplasmic Fractionation: Cells were lysed in 40 mM HEPES, pH 7.5, 5 mMEGTA, 0.1% Nonidet P-40, 5 mM MgCl2 1 mM DTT, 1 mM VO4, 1 mM benzinamide. The lysate was centrifuged (800g, 4 minutes) to obtain the cytoplasmic fraction as supernatant. Nuclei were resuspended in 50 mM β-glycerophosphate, pH 7.3, 0.2 mMEDTA, 420 mM NaCl, 1.5 mM MgCl2 1 mM DTT, 25% glycerol, sonicated briefly on ice, vortexed, and centrifuged (18000g, 10 minutes); and the precipitated cell debris was discarded.

Halo-TUBE pulldown: Cells were treated with either 2 mM DMOG, 10 μM MG132 or DMSO for 2 hours. After the treatments cells were washed in ice-cold PBS and lysed in 50 mM Tris·HCl, pH 7.5, 1 mM EGTA, 1 mM EDTA, 1% (vol/vol) Triton X-100, 0.27 M sucrose, 10 mM sodium 2-glycerophosphate, 1 mM sodium ortho-vanadate, 50 mM NaF, 5 mM sodium pyrophosphate, protease inhibitor cocktail and 10mM Iodoacetamide. Lysates were cleared by centrifugation, 0.1 vol was separated to be loaded as the total lysate, and the rest were incubated with TUBE beads rocking at 4°C for 16 h. Beads were collected and washed three times with ice cold lysis buffer +500mM NaCl. Pellets were resuspended in 5× Laemmli buffer, boiled for 5 min at 55ºC, and fractionated by SDS-PAGE.

Supplemental References:

Turriziani, B., Garcia-Munoz, A., Pilkington, R., Raso, C., Kolch, W., and von Kriegsheim, A. (2014). On-beads digestion in conjunction with data-dependent mass spectrometry: a shortcut to quantitative and dynamic interaction proteomics. Biology 3, 320-332.
