# Supplementary material for: PHD3 Regulates p53 Protein Stability by Hydroxylating Proline 359
Source: Cell Rep. 2018 Jul 31;24(5):1316–29. doi: 10.1016/j.celrep.2018.06.108 (PMC6088137; doi:10.1016/j.celrep.2018.06.108)
Supplement: Document S2. Article plus Supplemental Information [file mmc2.pdf]

Cell Reports

Article

PHD3 Regulates p53 Protein Stability by Hydroxylating Proline 359

Graphical Abstract

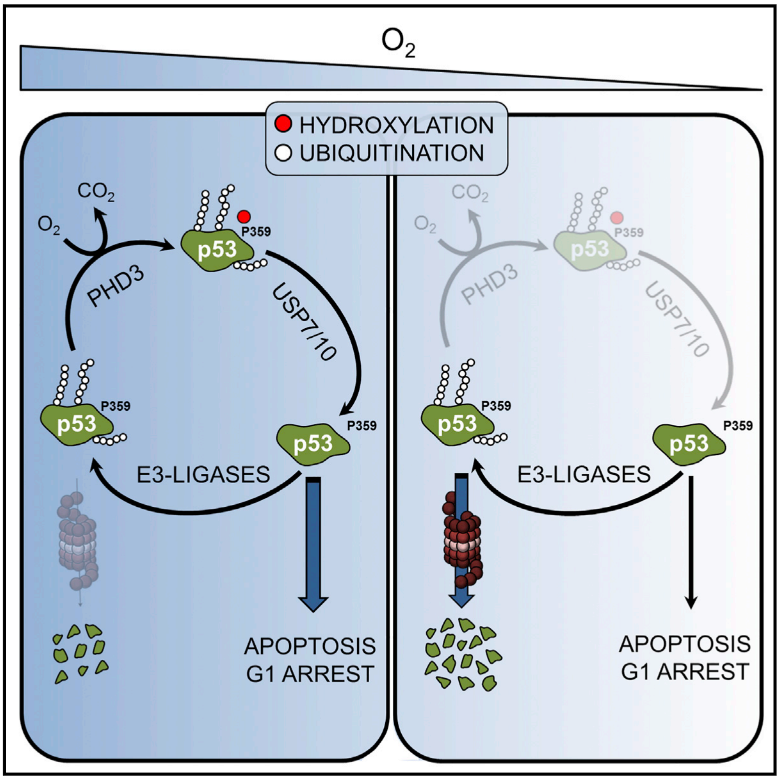

Highlights

- PHD3 hydroxylates p53 on Pro359
- Hydroxylated Pro359 forms a binding site for USP7/10
- Hydroxylase inhibition reduces USP binding and enhances p53 ubiquitination
- p53 protein stability and signaling regulated by PHD3 activity

Authors

Javier Rodriguez, Ana Herrero, Shuijie Li, ..., Cormac Taylor, Susanne Schlisio, Alex von Kriegsheim

Correspondence

alex.vonkriegsheim@igmm.ed.ac.uk

In Brief

It has been known for decades that hypoxia regulates p53 signaling. Rodriguez et al. here uncover a direct mechanism of how p53 protein stability is regulated by oxygen-sensing enzymes through a post-translational modification.

Rodriguez et al., 2018, Cell Reports *24*, 1316–1329
July 31, 2018 © 2018 The Author(s).
https://doi.org/10.1016/j.celrep.2018.06.108

OPEN ACCESS
CellPress

Cell Reports
Article

PHD3 Regulates p53 Protein Stability by Hydroxylating Proline 359

Javier Rodriguez,[1,5] Ana Herrero,[1] Shuijie Li,[3,4] Nora Rauch,[1] Andrea Quintanilla,[5] Kieran Wynne,[2] Aleksandar Krstic,[1] Juan Carlos Acosta,[5] Cormac Taylor,[2] Susanne Schlisio,[3,4] and Alex von Kriegsheim[1,5,6,*]
[1]Systems Biology Ireland, University College Dublin, Dublin 4, Ireland
[2]Conway Institute, University College Dublin, Dublin 4, Ireland
[3]Ludwig Institute for Cancer Research Ltd., SE-17177 Stockholm, Sweden
[4]Department of Microbiology and Tumor and Cell Biology, Karolinska Institutet, SE-17177 Stockholm, Sweden
[5]Cancer Research UK Edinburgh Centre, IGMM, University of Edinburgh, Edinburgh EH4 2XR, UK
[6]Lead Contact
*Correspondence: alex.vonkriegsheim@igmm.ed.ac.uk
https://doi.org/10.1016/j.celrep.2018.06.108

SUMMARY

Cellular p53 protein levels are regulated by a ubiquitination/de-ubiquitination cycle that can target the protein for proteasomal destruction. The ubiquitination reaction is catalyzed by a multitude of ligases, whereas the removal of ubiquitin chains is mediated by two deubiquitinating enzymes (DUBs), USP7 (HAUSP) and USP10. Here, we show that PHD3 hydroxylates p53 at proline 359, a residue that is in the p53-DUB binding domain. Hydroxylation of p53 upon proline 359 regulates its interaction with USP7 and USP10, and its inhibition decreases the association of p53 with USP7/USP10, increases p53 ubiquitination, and rapidly reduces p53 protein levels independently of mRNA expression. Our results show that p53 is a PHD3 substrate and that hydroxylation by PHD3 regulates p53 protein stability through modulation of ubiquitination.

INTRODUCTION

p53 is a potent tumor suppressor that functions as a stress-activated transcription factor regulating a multitude of cellular responses including apoptosis, senescence, DNA repair, and cell cycle arrest (el-Deiry et al., 1993; Levine, 1997; Li et al., 2012; Warboys et al., 2014). Oxygen deprivation or hypoxia is among the stresses that have been shown to induce p53 signaling leading to cell cycle arrest and apoptosis. The induction of p53 signaling appears to be dependent on the severity and duration of the oxygen deprivation (Alarcón et al., 1999; Graeber et al., 1994; Hammond and Giaccia, 2005; Koumenis et al., 2001). In addition to these dynamic regulations, in some cellular systems hypoxia has the diametrically opposed effect and decreases p53 protein levels (Chen et al., 2010; Sermeus et al., 2013). The reason why p53 responds to hypoxia in such a variable fashion is not yet resolved, and the same pathways have been used to explain both why p53 activity is induced or reduced by hypoxia. p53 activity, localization, and stability are tightly regulated by a variety of post-translational modifications including ubiquitination, acetylation, phosphorylation, and hydroxylation (Bode and Dong, 2004; Wang et al., 2014). Protein stability, as well as localization, are regulated by mono- and poly-ubiquitination mediated by ubiquitin ligases. Depending upon the cellular context, a variety of E3 ligases are thought to be the rate-liming factors in the regulation of p53 protein expression. One of these, MDM2, is regarded as the master regulator not only of p53 protein levels but also of p53 localization in a variety of cellular systems (Michael and Oren, 2003). Additionally, in cells infected with human papilloma virus (HPV), the viral E6 protein promotes the binding of p53 to the E3-ligase E6AP, which in turn ubiquitinates p53 efficiently and severely reduces cellular p53 protein levels (Talis et al., 1998).

Ubiquitination levels are not exclusively controlled by the forward reaction of the ligases. The modification is reversible and p53 is deubiquitinated by a family of proteases, the deubiquitinases (DUBs), two of which, USP7 (HAUSP) and USP10, regulate p53 (Li et al., 2002; Sheng et al., 2006; Yuan et al., 2010).

In addition to ubiquitination, several post-translational modifications including hydroxylations affect p53 signaling. Transcriptional activity is repressed by the hydroxylation of a C-terminal lysine catalyzed by JMJD6, a hydroxylase previously shown to act as histone demethylase and lysine hydroxylase (Wang et al., 2014). Three additional hydroxylases regulate the broader p53 pathway. FIH, an asparagine hydroxylase, hydroxylates the p53 binding protein ASPP2 on a C-terminal residue (Janke et al., 2013). Second, it was shown that PHD1, a proline hydroxylase, can regulate p53 by mediating the p38α-dependent phosphorylation of serine 15 in response to chemotherapeutic drugs (Deschoemaeker et al., 2015b). Finally, PHD3 enhances HCLK2 binding to ATR by hydroxylating several proline residues (Xie et al., 2012). PHD3 has been most prominently linked to the cellular response to hypoxia and is one of three PHDs shown to destabilize HIF1/2α (Epstein et al., 2001). We identified p53 as an interaction partner of PHD3 in a substrate-trap assay and were intrigued by this finding as regulation of p53 by PHD3 could help explain why p53 expression levels are regulated in a dynamic and non-linear fashion in hypoxic conditions. With this in mind, we set out to identify if and by which mechanism PHD3 directly regulates p53.

This is an open access article under the CC BY license (http://creativecommons.org/licenses/by/4.0/).

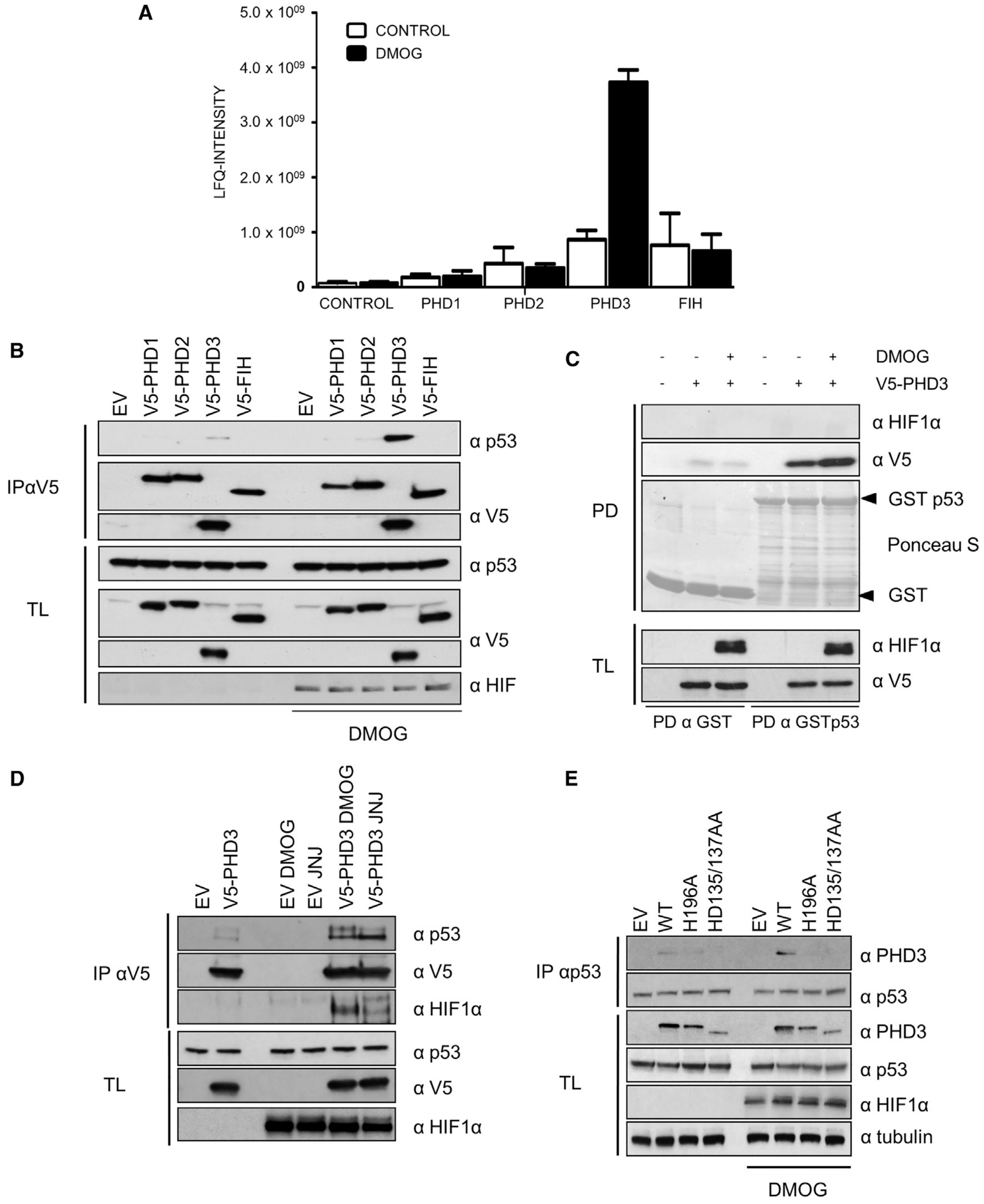

Figure 1. PHD3 Interacts with p53 in an Enzyme-to-Substrate-like Manner

(A) HEK293T cells were transfected in biological triplicates with an empty vector control, V5-tagged PHD1, PHD2, PHD3, or FIH. 24 hr post-transfection, the cells were treated with DMSO or DMOG for 4 hr. The V5-tagged proteins and their binding proteins were immunoprecipitated, digested, and analyzed by mass spectrometry. Bar graphs represent the LFQ intensities of p53 binding to the hydroxylases or a negative control. Error bars are SD, and n = 3.

(B) HEK293T cells were transfected with an empty vector control, V5-tagged PHD1, PHD2, PHD3, or FIH. 24 hr post-transfection, the cells were treated or not with DMOG for 4 hr. The cells were lysed (TL), and V5-tagged proteins and their binding proteins were immunoprecipitated (IP), separated on PAGE, electroblotted, and detected with the indicated antibodies.

(C) HEK293T cells were transfected with an empty vector control or V5-tagged PHD3, treated with DMOG for 4 hr or not, and lysed (TL). Cell lysates were incubated with bacterial expressed GST-p53 or GST bound to agarose beads for 2 hr. GST-pull-downs (PD) were washed, separated by PAGE, and electro-blotted, and proteins were detected by the indicated antibodies.

(legend continued on next page)

RESULTS

PHD3 Interacts with p53

Using quantitative mass spectrometry and a substrate "trapping" approach, we set out to identify substrates of HIF hydroxylases in HEK293T cells (Cockman et al., 2009; Rodriguez et al., 2016). Incubation with dimethyloxaloylglycine (DMOG), a pan-hydroxylase inhibitor, blocks the hydroxylase reaction and prevents the dissolution of the hydroxylase enzyme-substrate complex, increasing the enzyme-substrate complex. Quantifying dynamic, DMOG-dependent changes of the interactome allows for the distinction between generic interactors and likely substrates. We transfected HEK293T cells either with an empty vector or a V5-tagged hydroxylase and treated the cells with DMOG. Subsequently, the V5 immunoprecipitated proteins were identified and quantified by label-free quantification (LFQ) (Tate et al., 2013).

Using this approach, we detected the formation of a complex between PHD3 and p53 in normoxic conditions and additionally detected that this interaction was induced upon DMOG treatment (Figure 1A). We further confirmed the specificity of the interaction by immunoprecipitation and western blot (Figure 1B). These results suggested that p53 could be a novel substrate of PHD3. To determine whether this interaction could be reconstituted *in vitro*, we performed a glutathione S-transferase (GST) pull-down assay of recombinant V5-PHD3, using GST-p53 and GST as baits. We detected that p53 bound PHD3 and that incubation with DMOG increased the interaction, suggesting that p53 binds PHD3 directly (Figure 1C). In contrast, we were not able to reconstitute the postulated complex between HIF1α and p53 (Hansson et al., 2002) under our experimental conditions, probably related to a post-translational modification that is required on p53, which does not occur in our bacterial expression system.

To determine whether the induced interaction between p53 and PHD3 was not due to an off-target effect of DMOG, we compared the effect of the pan-hydroxylase inhibitor DMOG (which inhibits both PHDs and FIH) to the PHD-selective, structurally unrelated inhibitor JNJ-42041935 (JNJ) (Barrett et al., 2011). We observed that the p53/PHD3 complex was induced by both (Figure 1D). To analyze whether the active center of PHD3 was required for the interaction, we used two catalytically dead mutants of PHD3: H196A and HD135/137AA, which are mutated at the residues required for the correct iron binding (Bruick and McKnight, 2001). We immunoprecipitated endogenous p53 and observed that the interaction between p53 and PHD3 was absent in the case of both catalytically dead mutants (Figure 1E).

These results demonstrated that the interaction is direct and requires the presence of the intact prolyl hydroxylase active center, and inhibiting the hydroxylase with small chemical inhibitors "traps" the complex. These data suggest that the interaction between PHD3 and p53 is one of an enzyme to substrate.

p53 Stability Depends upon PHD3 Activity

HEK293T is a good cell model for analysis of protein-protein interactions as it maintains a high transfection efficiency even when using low amounts of DNA to allow for expression of tagged proteins close to physiological levels (Rodriguez et al., 2016). However, this cell line is not a good model of p53 signaling, as HEK293Ts express adenoviral oncoproteins e1a/e1b55k, which prevents p53 activation and degradation (Grand et al., 1999; Lowe and Ruley, 1993). Thus, we decided to analyze the effect of hydroxylase inhibition in two cell line models with wild-type (*wt*) p53 expression, but divergent regulation: HeLa cells, in which p53 degradation is mediated by the E6 oncoprotein (Hoppe-Seyler and Butz, 1993), and HepG2 cells, in which p53 stability is controlled by the action of MDM2 (Lu et al., 2000).

In HeLa cells, we observed that DMOG reduced endogenous p53 protein levels. This suppression could be rescued by MG132, an inhibitor of the 26S proteasome (Lee and Goldberg, 1998) (Figure 2A) and that p53 reduction was not limited to either nuclear or cytoplasmic localization (Figure S1A). We further showed that that hydroxylase inhibition had no effect on p53 mRNA levels in our experimental setup (Figure 2B), indicating that in HeLa cells the reduction of p53 levels by DMOG was mediated via proteasomal degradation. Additionally, to exclude that the reduction of p53 protein levels was due to the regulation of the translational machinery, we incubated the cells with cycloheximide (CHX), which has been shown to block the elongation phase of eukaryotic translation (Obrig et al., 1971). Pre-treating HeLa cells with DMOG reduced p53 protein half-life assayed by a CHX-chase experiment in HeLa cells (Figure 2C). These data suggest that hydroxylase inhibitors affect p53 protein levels by post-translational targeting for destruction via the proteasome.

Since hydroxylase inhibitors regulated p53 protein stability in HeLa cells, we wanted to determine whether endogenous PHD3 and p53 also interacted in these cells. We immunoprecipitated endogenous PHD3 or p53 and were able to detect p53 interacting with the PHD3 and vice versa (Figure 2D). Finally, we wanted to verify whether the reduction of p53 upon hydroxylase inhibition was mediated by PHD3. We transfected HeLa cells with either siNT (non-targeting small interfering RNA [siRNA]) or siPHD3 (siRNA specifically targeting PHD3) and observed that knockdown of PHD3 was sufficient to reduce p53 protein levels under normoxic conditions (Figure 2E). Under hypoxic conditions, cellular PHD3 activity can be reduced due to reduced oxygen tension or, counterintuitively, enhanced because of the strong induction of PHD3 protein expression. This non-linear behavior of global PHD3 activity in hypoxia may reduce or enhance p53 protein levels depending on the cellular and environmental context. To see whether we could detect such non-linearity, we incubated HeLa cells in DMOG or 1% oxygen for up to 24 hr, by which time PHD3 protein levels are induced. Upon DMOG treatment, p53 levels quickly decrease and recover

(D) HEK293T cells were transfected with an empty vector control or V5-tagged PHD3. 24 hr post-transfection, the cells were treated with DMSO, DMOG, or JNJ for 4 hr. The cells were lysed, and V5-tagged proteins and their binding proteins were immunoprecipitated, separated on PAGE, electroblotted, and detected with the indicated antibodies.

(E) HEK293T cells were transfected with an empty vector or PHD3 *wt*, H196A, or HD135/137AA. 24 hr post-transfection, the cells were treated or not with DMOG for 4 hr. Endogenous p53 was immunoprecipitated, separated on PAGE, and electroblotted, and proteins were detected with the indicated antibodies.

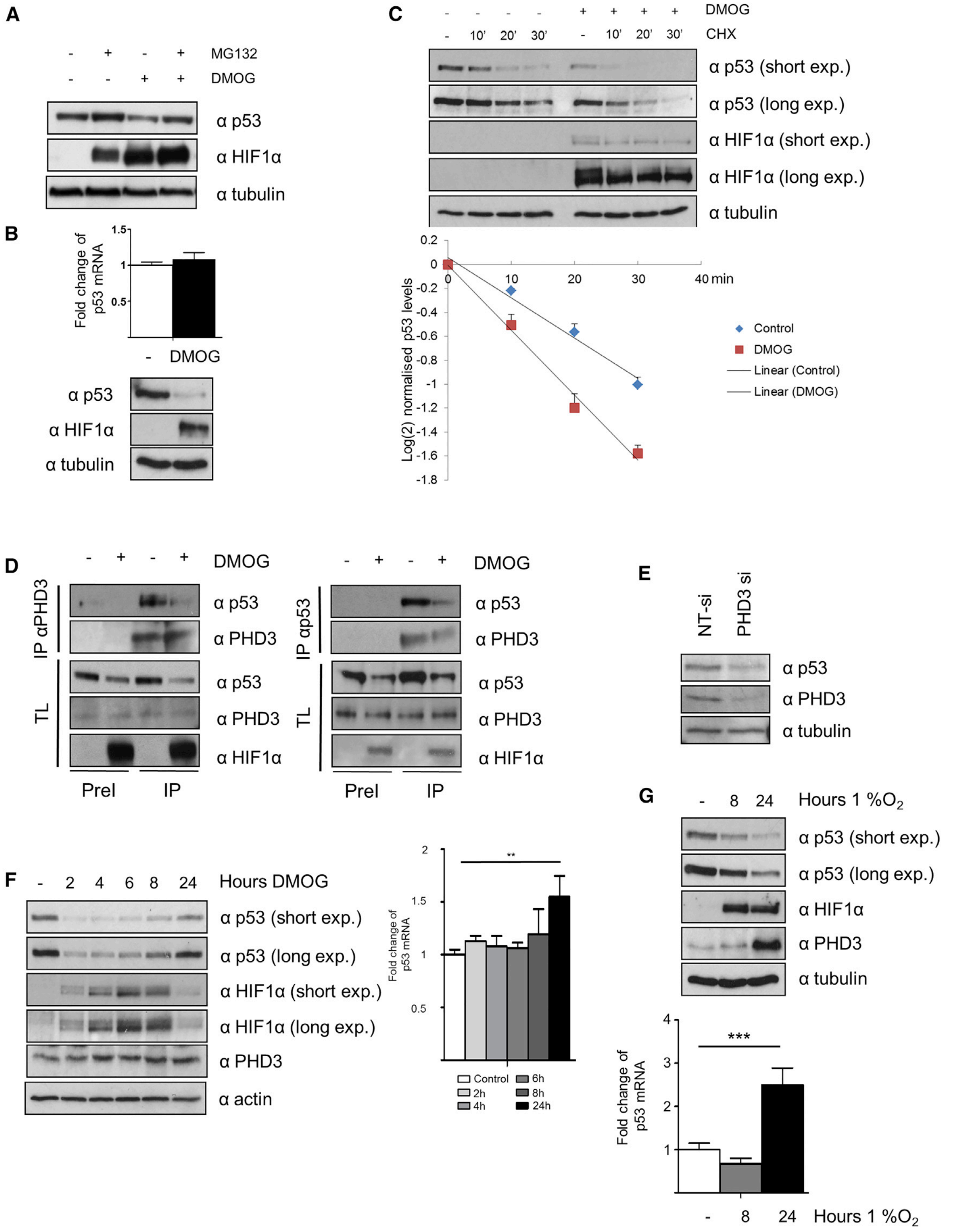

Figure 2. PHD3 Regulates p53 Protein Stability

(A) HeLa cells were treated with DMOG for 4 hr in the presence or absence of MG132. Cells were lysed, and proteins were separated by PAGE, electroblotted, and detected by the indicated antibodies.

(B) HeLa cells were treated with DMSO or DMOG, and after 4 hr cells were harvested for quantitative RT-PCR assays. The expression level of p53 was normalized to endogenous actin mRNA levels. The values plotted are means ± SD of N = 3 independent experiments for each condition. In parallel, a western blot was performed to validate DMOG treatment.

(legend continued on next page)

at later time points. We observed an induction of p53 mRNA; we cannot therefore exclude that the induction of p53 protein levels is due to enhanced transcription (Figure 2F). In hypoxia, to our surprise, we could not detect p53 levels rebounding, despite an increase in the underlying p53 mRNA signal, suggesting that in this cell line PHD3 activity is suppressed in 1% oxygen (Figure 2G). Overexpression of exogenous PHD3 in HeLa cells did not reproducibly increase p53 levels, but re-expression of exogenous PHD3 rescued p53 protein levels following PHD3 knockdown (Figures S1B and S1C).

We went on to perform similar experiments in HepG2 cells, in which MDM2 is the main regulator of p53 stability. We analyzed the effect of two structurally distinct hydroxylase inhibitors and hypoxia on p53 protein levels and observed that hydroxylase inhibition led to the reduction of p53 levels. We observed that MG132 blocked the reduction of p53 triggered by DMOG or JNJ (Figure 3A) and further confirmed that the reduction of the p53 protein levels was not due to a suppression of mRNA levels (Figures 3B, S2A, and S2B). In light of this result, we assayed p53 protein stability in HepG2 cells and determined that p53 protein half-life was reduced in the presence of DMOG (Figure 3C). To check whether this was mediated by PHD3 activity, we knocked down PHD3 and monitored p53 expression. PHD3 knockdown was sufficient to reduce p53 levels under normoxic and hypoxic conditions (Figure 3D). This reduction in p53 levels was linked with an increase of the proteasomal degradation as the treatment with the proteasomal inhibitor MG132 rescued this reduction (Figure 3E). We could not observe that prolonged hypoxia or DMOG treatment led to p53 levels rebounding together with increasing PHD3 levels and that overexpression of PHD3 did not significantly stabilize p53 level in normoxia (Figures S2A–S2C). We could nevertheless rescue p53 expression levels by re-expressing exogenous PHD3 in cells where PHD3 had been knocked down by siRNA (Figure S2D).

An increase in proteasomal degradation is associated with ubiquitination of the protein. To determine whether hydroxylase inhibition increased p53 ubiquitination, we precipitated ubiquitinated proteins by tandem ubiquitin binding entities (TUBEs) pull-downs in HepG2 cells incubated with or without DMOG and MG132. We detected that DMOG increased p53 ubiquitination compared to the untreated sample (Figure 3F). These results suggest that hydroxylase inhibitors and PHD3 specifically promote a reduction in p53 levels via an ubiquitination-dependent proteasomal mechanism.

p53 Hydroxylates PHD3 at Proline 359

Having determined that the activity of PHD3 is required for p53 stability, we tested whether p53 can be directly hydroxylated by PHD3. Initially, we performed an *in vitro* hydroxylation assay with HEK293T lysate overexpressing V5-PHD3 in conjunction with recombinant GST-p53 as substrate and detected hydroxylated prolines by liquid chromatography-tandem mass spectrometry (LC-MS/MS). In total, we were able to detect nine hydroxyprolines in GST-p53 after the *in vitro* hydroxylation reaction (Figures S3A–S3I). Following on from this exploratory analysis, we determined whether any of these hydroxylation sites could be detected *in vivo* and were regulated by PHD3 activity.

Using Flag-p53 as substrate, we performed an *in vivo* hydroxylation assay in which we utilized two opposing extreme conditions. In one, we induced hydroxylation levels by overexpressing V5-PHD3, and in the other we suppressed levels of protein hydroxylation by treating the cells with DMOG. In a comparative analysis between these two experimental conditions, we were only able to identify one of the previously detected hydroxyprolines (Figure S4A) and observed a reduction of this hydroxylation upon DMOG treatment. The hydroxylation site identified was located at proline 359, which lies in the C-terminal domain of p53 (Figure 4A). Additionally, in order to confirm that PHD3 is one of the enzymes that promote the hydroxylation of Pro359 in an oxygen-dependent manner, we analyzed the effect of PHD3 siRNA and hypoxia on the hydroxylation levels. We observed that the hydroxylation of P359 decreased in cells transfected with PHD3 siRNA and that 1% oxygen reduced hydroxylation levels below the limit of detection, supporting the concept that Pro359 is a target of a PHD3 and oxygen-dependent hydroxylation (Figure 4B).

To confirm that PHD3 directly hydroxylates P359, we incubated a biotinylated peptide (amino acids 349–371 of human p53) with extracts of HEK293T cells overexpressing PHD3 wt or the inactive mutant PHD3 H196A and observed an increase of proline 359 hydroxylation when the peptide was incubated with PHD3 wt in comparison to the inactive hydroxylase. As an internal control, the oxidation levels of the biotin residue were also monitored, which showed no difference (Figures 4C and S4B). Last, we incubated purified, recombinant PHD3 with the peptide and could confirm the hydroxylation (Figure S4C).

To demonstrate that PHD3 interacts with the peptide, we incubated lysates overexpressing PHD3 wt or the inactive mutant with the peptides. We detected the interaction of PHD3 wt with

(C) HeLa cells were incubated with DMOG or DMSO for 4 hr and treated afterward with/without CHX (10 μg/mL) for up to 30 min. Cells were lysed, and proteins were separated by PAGE, electroblotted, and detected by the indicated antibodies. p53 intensity normalized to the respective (DMOG or DMSO) 0-time point were plotted on an xy diagram with log(2)-transformed x-axis to visualize protein half-life.
(D) HeLa cells were treated with or without DMOG for 4 hr. The cells were lysed (TL) and endogenous PHD3 or p53 was immunoprecipitated (IP), using a pre-immune antibody as negative control (PreI). Total lysates and the corresponding immunoprecipitates were probed for the indicated proteins for immunoblotting.
(E) HeLa cells were transfected with non-targeting (NT) or PHD3-specific siRNA. 48 hr post-transfection, the cells were treated with DMOG and lysed, and proteins were separated by PAGE, electroblotted, and detected by the indicated antibodies.
(F) HeLa cells were treated with DMOG as indicated and lysed, and proteins were separated by PAGE, electroblotted, and detected by the indicated antibodies. In parallel, mRNA was extracted and quantified by RT-PCR (second panel).
(G) HeLa cells were cultured in 1% oxygen as indicated and lysed, and proteins were separated by PAGE, electroblotted, and detected by the indicated antibodies. In parallel, mRNA was extracted and quantified by RT-PCR (second panel). The values plotted are means ± SD of N = 3 independent experiments for each condition.
Two-tailed, equal distribution Student's t test was employed to test for statistical difference with values of $^{**}p < 0.01$ and $^{***}p < 0.001$

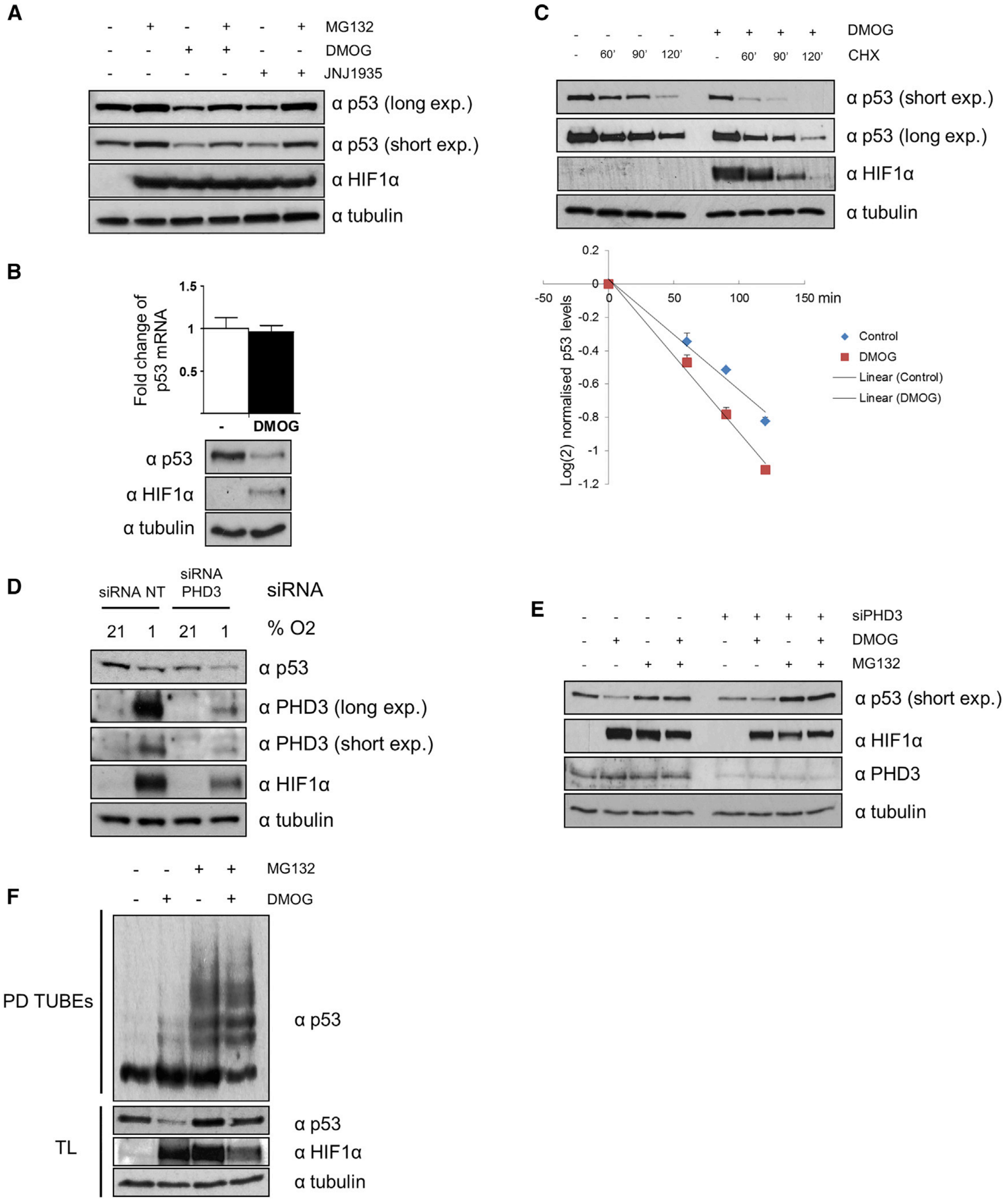

Figure 3. PHD3 Regulates p53 Protein Stability and Ubiquitination

(A) HepG2 cells were treated with DMOG of JNJ for 4 hr in the presence or absence of MG132. Cells were lysed, and proteins were separated by PAGE, electroblotted, and detected by the indicated antibodies.

(B) HepG2 cells were treated with DMOG, and after 4 hr, cells were harvested for quantitative RT-PCR assays. The expression level of p53 was normalized to endogenous actin mRNA levels. The values plotted are means ± SD of N = 3 independent experiments for each condition. In parallel, a western blot was performed to validate DMOG treatment.

(C) HepG2 cells were incubated with DMOG or DMSO for 2 hr and treated afterward with/without CHX (10 μg/mL) for up to 120 min. Cells were lysed, and proteins were separated by PAGE, electroblotted, and detected by the indicated antibodies. p53 intensity normalized to the respective (DMOG or DMSO) 0-time point was plotted on an xy diagram with log(2)-transformed x-axis to visualize protein half-life.

(D) HepG2 cells were transfected with non-targeting (NT) or PHD3-specific siRNA. 24 hr post-transfection, the cells were placed in a hypoxia chamber (Coy Laboratories, Grass Lake, MI) during 24 hr (1% O_2, 5% CO_2, and 94% N_2). Normoxic controls were maintained at atmospheric O_2 levels (21% O_2, 5% CO_2, and

(legend continued on next page)

the peptide but were unable to detect the interaction of PHD3 H196A (Figure 4D). To determine that proline 359 was essential for the binding of the hydroxylase with the peptide, we synthesized the analogous peptide exchanging proline 359 for hydroxyproline. We then incubated the proline- and hydroxyproline-containing peptides with lysates overexpressing PHD3 and observed that hydroxylation of proline 359 reduced the binding of the peptide to PHD3 (Figure 4E).

In light of these results, we tested whether the DMOG-induced reduction of p53 protein levels required the presence of proline 359 in p53. We generated a mutant of p53, where proline was exchanged for alanine (P359A), and expressed the Flag-tagged mutant and wt p53 in HepG2. We readily detected the reduction of Flag-p53 wt and of the endogenous protein (Figure 4F). P359A mutant levels were not reduced by DMOG treatment. Proline-to-alanine mutations can alter the secondary structure of the protein; therefore, to ascertain that the insensitivity of the P359A mutant was not due to an unspecific disruption of the structure, we mutated two neighboring proline to alanine (P316/318A) and repeated the assay. In contrast to the P359 mutation, p53 P316/318A protein expression behaved analogously to the wt protein (Figure 4G). These results show that PHD3 hydroxylates p53 at the Pro359 and that the hydroxylation of this residue regulates p53 stability.

As mentioned previously, PHDs regulate upstream effectors of the p53 signaling network, which manifests in the modification of p53 phosphorylation on serine 15. To ascertain that phosphorylation of this residue is not causal to the reduction of p53 protein levels upon hydroxylase inhibition, we mutated this residue to alanine (S15A). We repeated the assay and observed that p53 S15A expression was also reduced by DMOG treatment (Figure 4H), demonstrating that this downregulation of p53 is independent of S15 phosphorylation.

Hydroxylation of P359 Regulates the Binding of p53-USP7/10

Having demonstrated P359 hydroxylation and consequent regulation of p53 stability, we pursued approaches that would reveal the underlying molecular mechanism. Proline hydroxylation generally affects protein-protein interactions. Consequently, we used an unbiased approach to determine how hydroxylase inhibitors affected the interactome of exogenous Flag-p53. Using a quantitative mass spectrometry approach and focusing on ubiquitin ligases and proteases, we were able to determine that the interaction between p53 and two DUBs, USP7 and USP10, was reduced under DMOG treatment (Figure 5A). USP7 interacts and regulates p53 as well as MDM2 stability by promoting their deubiquitination (Li et al., 2002; Ma et al., 2010). The interaction of USP7 with p53 is well characterized, and amino acids 359–367 have been identified as responsible for p53 binding to USP7 (Sheng et al., 2006). USP10 also promotes the deubiquitination of p53 (Yuan et al., 2010), although the precise interaction domain within p53 has not yet been mapped. To confirm our mass spectrometry data by alternative means, we immunoprecipitated exogenous Flag-tagged USP7 in HEK293T cells and observed that DMOG reduced the binding between Flag-USP7 and endogenous p53 (Figure 5B). To further confirm that the interaction required Pro359 hydroxylation, we expressed Flag-USP7, His p53, and His p53 P359A in HEK293T cells and immunoprecipitated His-tagged p53 in the presence or absence of DMOG. We observed that DMOG and the mutation of Pro359 strongly reduced the interaction between p53 and USP7 (Figure 5C).

To confirm the hydroxylation-dependent regulation of the p53-USP10 interaction, we performed an analogous experiment. Flag-p53 wt and Flag-p53 P359A were transfected into HEK293T cells and immunoprecipitated. Similar to what we observed with USP7, inhibition of hydroxylase activity diminished the interaction between p53 and endogenous USP10. Furthermore, P359A mutation reduced the interaction with USP10 in comparison to p53 wt and the interaction between the p53-P359A and USP10 was not regulated by DMOG (Figure 5D). To unequivocally determine that hydroxylation of P359 enhances binding to USP, we incubated peptides containing proline or hydroxyproline 359 with lysates overexpressing USP7 or USP10 and observed that hydroxylation of proline 359 increased the binding of the peptide to both USPs (Figure 5E). We additionally confirmed the interaction endogenously by immunoprecipitating USP10 from HepG2 cells treated with DMOG or not and detecting co-purifying p53 (Figure S5A). To determine whether the absence of a USP7/10 binding site on p53 increased p53 ubiquitination, we transfected Flag-p53 wt and Flag-p53 P359A into HepG2 cells and precipitated ubiquitinated proteins by TUBE pull-downs. We detected that the P359A mutant had higher ubiquitination levels when compared to the wt (Figure S5B).

These results show that DMOG reduces the interaction between p53 and USP7/10, Pro359 is required for binding with both USPs, mutation of Pro359 to alanine eliminates the DMOG sensitivity of the interaction, and hydroxylation of Pro359 enhances p53-USP7/10 binding.

To establish whether the reduction of p53 under hydroxylase inhibition was due to reduced DUB activity or binding, we transfected HepG2 cells with siNT or siRNA targeting USP10 (siUSP10). In accordance with published work, we detected that knockdown of USP10 is sufficient to reduce p53 protein levels (Yuan et al., 2010). Treatment of the knockdown cells with DMOG did not further reduce p53 levels (Figure 5F). In summary, these results demonstrate that the loss of hydroxylation of Pro359 reduces the interaction between p53 and USP7/10. In

74% N_2) in a tissue culture incubator. After this time, the cells were lysed and proteins were separated by PAGE, electroblotted, and detected by the indicated antibodies.

(E) HEPG2 cells were transfected with non-targeting (NT) or PHD3-specific siRNA. 48 hr post-transfection, the cells were lysed, and proteins were separated by PAGE, electroblotted, and detected by the indicated antibodies.

(F) HepG2 cells were treated with DMOG and/or MG132 for 2 hr. Cells were lysed and ubiquitinated proteins were precipitated with TUBE-agarose (PD). Proteins were separated by PAGE and electroblotted. Ubiquitinated p53 was detected by a specific antibody (PD), and changes in expression in total lysate were blotted separately (TL).

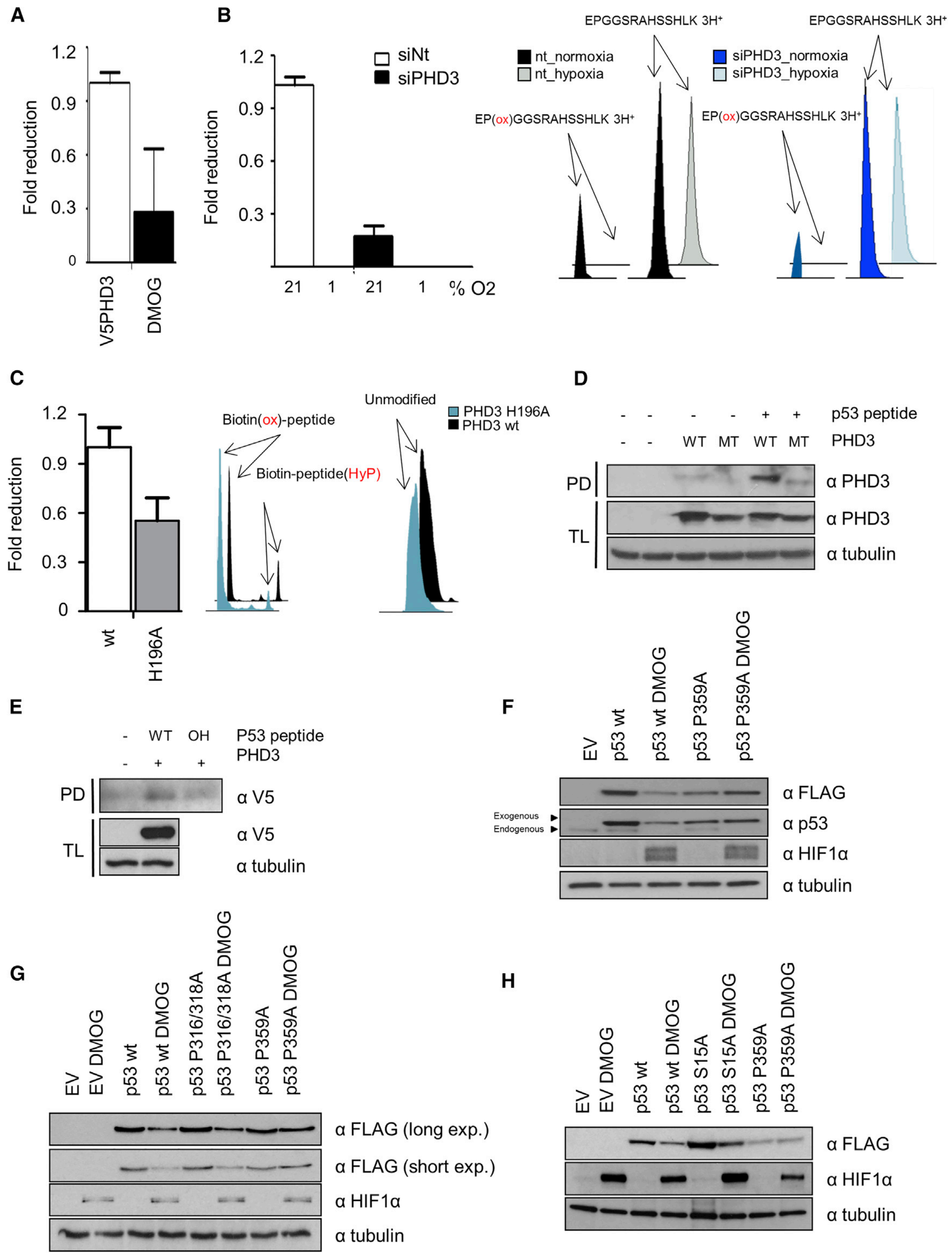

Figure 4. p53 Is Hydroxylated on Pro359 by PHD3

(A) HEK293T cells were transfected with Flag-p53, an empty vector control, or V5-tagged PHD3. 24 hr post-transfection, the cells were treated with DMSO or DMOG for 4 hr. Flag-p53 was immunoprecipitated, digested with Lys-C, and analyzed by mass spectrometry. Bar graph represents the normalized hydroxylation ratio of p53 peptide. Error bars represent SEM, and n = 2.

(legend continued on next page)

addition, we show that loss of USP10 mimics the effects of hydroxylase inhibition and that hydroxylase inhibition and USP10 knockdown are not additive, thus likely part of the same mechanism regulating p53 protein stability under these circumstances.

Reduction of p53 Hydroxylation Reduces p53 Signaling

p53 is a key regulator of antiproliferative and apoptotic responses. In light of our data demonstrating the PHD3-dependent regulation of p53 via hydroxylation of P359, we hypothesized that inhibition or knockdown of PHD3 may regulate signaling downstream of p53. PHD3 has been shown to promote p53-mediated apoptosis in response to DNA-damaging agents by hydroxylating HCLK2 and activating S15 phosphorylation on p53 (Xie et al., 2012). In light of this report, we decided not to induce p53 expression or activity through DNA damage or other stress responses as it would be impossible to separate the effect mediated by P359 hydroxylation from upstream events.

Working within these constraints, we determined whether DMOG by itself affected p53 transcriptional activity by observing expression levels of p21, a prominent p53 effector (el-Deiry et al., 1994; Sheikh et al., 1994). In HepG2 cells, DMOG not only reduced p53 levels but also markedly reduced p21 expression (Figure 6A).

To determine whether the hydroxylase-dependent reduction of p53/p21 affected systemic p53 signaling, we analyzed whether expression and activity of PHD3 affected p53-induced cell cycle arrest. To avoid crosstalk from stress-response pathways, we induced p53 and cell cycle arrest with nutlin-3, a chemical inhibitor of MDM2-dependent degradation. Nutlin-3 stabilized p53 protein levels but was not able to rescue the reduction of p53 in response to PHD3 knockdown or inhibition, confirming that the functional MDM2 and PHD3 act independently of each other (Figure S6A). Nutlin-3 treatment was sufficient to stabilize p53 and increase the percentage of cells in G1 (Vassilev, 2004) in HepG2 cells. As reported, knockdown of PHD3 increased cells in G1 (Högel et al., 2011). Nutlin-3 stabilized p53 and increased the G1 population. PHD3 knockdown partially rescued p53 stabilization and the proportional increase of cells in G1 (Figures 6B, 6C, and S6B).

An additional model to study the role of p53 independent of upstream stress pathways is the previously characterized doxycycline (DOX)-inducible p53 Saos-2 cell line (Nakano et al., 2000). We initially tested whether hydroxylase inhibitors affected the DOX induction of p53 mRNA. JNJ did not affect the DOX-dependent induction of p53 mRNA levels, whereas DMOG severely reduced them, likely through an off-target effect on the DOX induction (Figure S6C). In light of these data, we decided to proceed with JNJ alone in this cell line.

JNJ reduced p53 levels (Figure 6D). We further confirmed that reduction of PHD3 expression by siRNA was sufficient to reduce p53 levels (Figure 6E). Suppression of p53 coincided with a reduction of p21 upon hydroxylase inhibition and PHD3 knockdown. In this inducible model, it has been shown that induction of p53 by DOX robustly induces apoptosis. Using YO-PRO1 as a marker of apoptosis, we observed that p53 induction by DOX-induced apoptosis, which could be partially rescued by PHD3 knockdown (Figures 6F and S6D).

Overall, we could confirm that inhibition or knockdown of PHD3 reduced p53 and p21 levels in both model systems. Given the clear effects on p53 levels and activity, it is perhaps surprising that the effects of PHD3 inhibition on p53-dependent cell cycle arrest and apoptosis are not stronger. However, it is likely that PHD3 inhibition affects cell cycle and survival pathways, which may limit the magnitude of the observed rescues.

PHD3 expression is frequently attenuated in high-grade gliomas (Henze et al., 2014), and we hypothesized that re-expression of the active PHD3 may stabilize p53 and impede cell proliferation and apoptosis in cell lines where PHD3 is silenced. To test this hypothesis, we re-expressed PHD3 in U87, a glioma cell line where PHD3 is silenced (Sciorra et al., 2012). To control that the effects of PHD3 re-expression were mediated by p53, we also infected the cells either with a scrambled or p53-specific short hairpin RNA (shRNA). Re-expression of wt PHD3 but not the inactive mutant by adenoviral infection led to a stabilization of p53 and cleavage of caspase 3 (Figure 6G). Knocking down p53 impaired caspase cleavage, indicating that PHD3-induced caspase cleavage required p53. In addition, re-expression of PHD3 impeded U87 cell proliferation, which was again partially

(B) HEK293T cells were transfected with Flag-p53 in the presence of a non-targeting (NT) or PHD3-specific siRNA. 48 hr post-transfection, the cells were cultured in hypoxia for 24 hr. Flag-p53 was immunoprecipitated, digested with LysC, and analyzed by mass spectrometry. Bar graphs represent the normalization of the ratio modified/unmodified peptide intensities. Error bars represent SEM, and n = 2. XIC of EP(ox)GGSRAHSSHLK and non-hydroxylated EPGGSRAHSSHLK.
(C) Biotinylated peptides ELKDAQAGKEPGGSRAHSSHLKS were incubated with lysates derived from HEK293T cells transiently transfected with PHD3 wt or inactive mutant H196A. Bar graphs represent the ratio of the intensities of the modified and unmodified peptide. Error bars represent SEM, and n = 2. XIC of biotin(ox)-ELKDAQAGKEPGGSRAHSSHLKS (left peak) and biotin-ELKDAQAGKEP(ox)GGSRAHSSHLKS (blue) and non-hydroxylated ELKDAQAG KEPGGSRAHSSHLKS (black).
(D) HEK293T cells were transfected with the indicated PHD3 plasmids (ev, HA-PHD3 wt or PHD3 H135A/D137A). Pull-down was performed using as a bait P359 peptide. P359A peptide was bound previously to streptavidin agarose beads, and streptavidin agarose beads were used as a negative control. Pull-downs and the corresponding total lysates were tested by western blot for the indicated proteins.
(E) HEK293T cells were transfected with empty vector or V5-PHD3 plasmid. The cellular lysate, overexpressing V5-PHD3, was split in two for further pull-downs with the two different peptides. Pull-down was performed using as a bait different P359 peptides (*wt* and hydroxylated at the proline 359). These peptides were bound previously to streptavidin agarose beads. Streptavidin agarose beads were used as a negative control. Pull-downs and the corresponding total lysates were tested by western blot for the indicated proteins.
(F) HepG2 cells were transfected with Flag-p53 wt or P359A mutant. 24 hr post-transfection, the cells were treated with DMOG for 4 hr. Total lysates were separated on PAGE, electroblotted, and detected with the indicated antibodies.
(G) HepG2 cells were transfected with *wt* and P359A and P316/318A mutants of Flag-p53. After 24 hr, the cells were treated with DMSO or DMOG for 4 hr, and the corresponding total lysates were tested by western blot for the indicated proteins.
(H) HEK293T cells were transfected with empty vector (control), Flag-p53 *wt*, Flag-p53 S15A, or Flag-p53 P359A for 24 hr. Following this time, cells were treated with DMOG or DMSO for 4 hr. Total lysates were analyzed by western blotting for the indicated proteins.

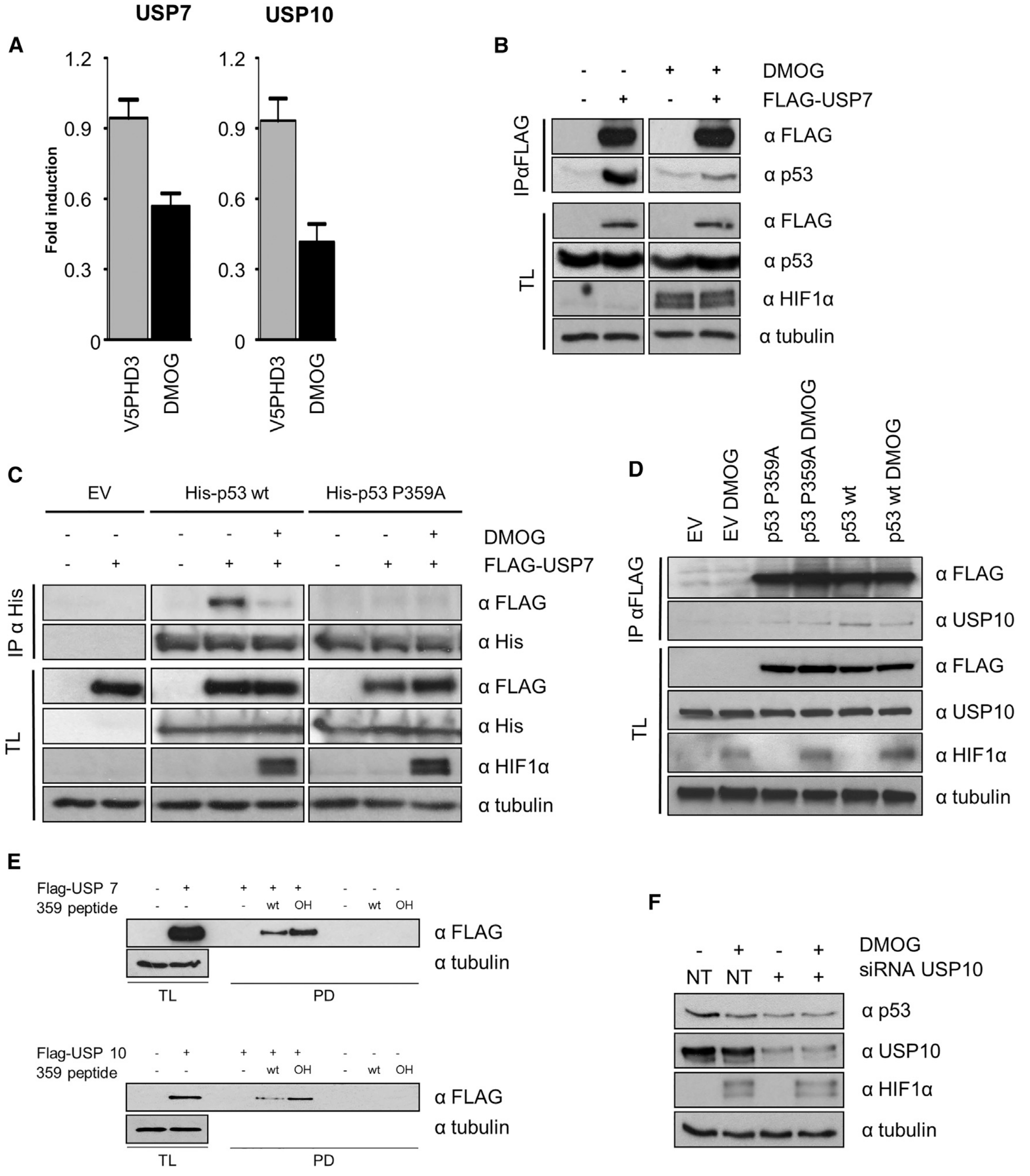

Figure 5. p53 Hydroxylation on Pro359 Regulates the Binding of USP7/10

(A) HEK293T cells were transfected with Flag-p53. 24 hr post-transfection, the cells were treated with DMSO or with DMOG for 4 hr. Flag-p53 was immunoprecipitated, digested with trypsin, and analyzed by mass spectrometry. Bar graphs represent the normalized LFQ intensities of USP7 or USP10 co-precipitating with p53. Error bars are SD, and n = 2.

(B) HEK293T cells were transfected with Flag-USP7. 24 hr post-transfection, the cells were treated with DMSO or DMOG for 4 hr. Immunoprecipitation with anti-Flag beads was followed by western blot analysis with the indicated antibodies.

(C) HEK293T cells were transfected with Flag-USP7 and/or wt or P359A mutant His-tagged p53. 24 hr post-transfection, the cells were treated or not with DMOG for 4 hr. His-tagged proteins were immunoprecipitated. Proteins were separated by PAGE, electroblotted, and detected with the indicated antibodies.

(D) HEK293T cells were transfected with Flag-p53 wt or P359A mutant. 24 hr post-transfection, the cells were treated or not with DMOG for 4 hr. Immunoprecipitation with anti-Flag beads was followed by western blot analysis with the indicated antibodies.

(E) HEK293T cells were transfected with empty vector or Flag-tagged USP7 or 10 plasmids. The cellular lysate, overexpressing USPs, was split in three for further pull-downs with the two different peptides and a streptavidin-only control. 1% of the volume was kept as total lysate. Pull-down was performed using as a bait different P359 peptides (*wt* and hydroxylated at the proline 359). These peptides were bound previously to streptavidin agarose beads.

(legend continued on next page)

rescued by knocking-down p53 (Figure 6H). Third, transient transfection of PHD3 robustly induced cellular DNA fragmentation, which was diminished upon p53 knockdown (Figures 6I and S6E).

These data confirm our hypothesis that PHD3 regulates p53 stability and that the reported antiproliferative/proapoptotic functions of PHD3 are partially mediated by p53. Nevertheless, despite a near-complete knockdown of p53, PHD3 expression still exhibited pronounced pro-apoptotic and anti-proliferative effects, indicating that only a part of the PHD3-dependent stress signaling requires p53. These data are in line with previous findings demonstrating that PHD3 elicits apoptotic or antiproliferative signaling independent of p53 through KIF1Bβ, by modulating EGFR signaling, through acetyl-coA carboxylase 2 or by inducing protein aggregates (German et al., 2016; Henze et al., 2014; Lee et al., 2005; Rantanen et al., 2008; Schlisio et al., 2008).

DISCUSSION

It has been known for decades that hypoxia and hydroxylase inhibition regulate expression and activity of p53. Nevertheless, the direction and amplitude of the regulation appear to be controversial, with many studies reporting an increase, a decrease, or no effect on protein levels at all (Pan et al., 2004). At first sight, these results appear to be contradictory, but given the highly complex regulation of p53 by a multitude of translational, post-translational, and feedback regulations, this should not be surprising. We can observe these discrepancies in our own data. p53 levels are transiently reduced in HeLa cells upon DMOG treatment, whereas HepG2 respond in a sustained manner. We assume that the combinatorial effect of PHD3, p53, oxygen, and tricarboxylic acid (TCA) cycle intermediates all play a role in shaping the response. As an example, there are data suggesting that part of stress signaling following hypoxia/reoxygenation cycles is dependent on p53 (Gogna et al., 2012; Weinmann et al., 2004). We hypothesized that high levels of PHD3 combined with normoxic oxygen concentration upon reoxygenation could lead to the rapid stabilization of p53, priming the stress response. We tested this hypothesis in HepG2 cells. 24 hr in hypoxia induced PHD3 and reduced p53 as well as p21 protein levels (Figures 6J and S6F). We observed that, upon reoxygenation, p53 and p21 were stabilized within 1 hr. This stabilization was PHD3 dependent as knockdown of PHD3 not only reduced p53 levels in normoxia, but also prevented the induction of p53 and p21 upon reoxygenation. Nevertheless, it is obvious that hydroxylation of p53 on P359 is only a part of the overall picture. Hydroxylases themselves mediate p53 activity, stability, and localization at multiple levels (Deschoemaeker et al., 2015a; Janke et al., 2013; Wang et al., 2014; Xie et al., 2012), and recently the PHD1-dependent hydroxylation of p53 has been postulated (Ullah et al., 2017). Such distributed controls are common in most signaling pathways, and it is emerging that the HIF pathway itself is regulated by hydroxylation at multiple levels. Apart from enabling the VHL-HIF1/2α interaction (Ivan et al., 2001; Jaakkola et al., 2001), hydroxylases regulate the HIF1/2α interaction with p300 through hydroxylation of a C-terminal residue (Hewitson et al., 2002). In addition, it was recently shown that PHD1 can hydroxylate and activate DYRK1 kinases, which phosphorylate ID2 and prevent the disruption of the VHL degradation complex, which in turn destabilizes HIF2α (Lee et al., 2016). The realization of the increasing complexity with numerous feedforward and feedback loops can already explain some ostensibly contradictory reports. This highlights the necessity of doing experiments with sufficient temporal resolution across multiple model systems and, if possible, to generate mathematical models based on these data.

EXPERIMENTAL PROCEDURES

Immunoblotting

Total lysates and affinity precipitates were fractionated by SDS-PAGE and transferred onto nitrocellulose filters. Immuno-complexes were visualized by enhanced chemiluminescence detection (GE Healthcare) with horseradish peroxidase-conjugated secondary antibodies (Bio-Rad Laboratories). Experiments were repeated at least three times.

Mass Spectrometry

Cells were transfected with empty vector, a V5-tagged hydroxylase, or Flag-tagged p53, and treated 24 hr post-transfection with either 2 mM DMOG or DMSO for 4 hr. The cells were lysed, and we immunoprecipitated the protein with anti-V5 or anti-Flag agarose for 2 hr. The samples were digested with trypsin or Lys-C and processed as previously described (Turriziani et al., 2014). Desalted peptides were analyzed on a Fusion Lumos mass spectrometer (Thermo, Germany). Experiments consisted of three biological replicates or as indicated.

Statistical Analysis

Two-tailed, equal distribution Student's t test was employed to test for statistical difference with values of $*p < 0.05$, $**p < 0.01$, and $***p < 0.001$.

SUPPLEMENTAL INFORMATION

Supplemental Information includes Supplemental Experimental Procedures and six figures and can be found with this article online at https://doi.org/10.1016/j.celrep.2018.06.108.

ACKNOWLEDGMENTS

We thank Bob Ludwig for sending the SAOS-p53 cell lines, Cynthia Coffill for Flag-p53, Greg Semenza via Till Acker for HD135/137AA PHD3 from Greg Findlay, Francisco Bustos for HALO-TUBEs, Alfonso Blanco for assistance with FACS, and Arek Welman and Andrew Finch for critical reading, as well as the following funding agencies: Science Foundation Ireland (13/SIRG/2174), Cancer Research UK (CRUK Edinburgh Centre C157/A25140), Wellcome Trust (Multiuser Equipment Grant 208402/Z/17/Z), and Carnegie Trust (RIG, 70382).

AUTHOR CONTRIBUTIONS

J.R., C.T., and A.v.K. designed the experiments and interpreted the results. J.R. performed most of the experiments. A.H. performed FACS analysis and

Streptavidin agarose beads were used as a negative control. Pull-downs and the corresponding total lysates were tested by western blot for the indicated proteins.

(F) HepG2 cells were transfected with non-targeting (NT) or USP10-specific siRNA. 48 hr post-transfection, the cells were treated with DMSO or DMOG for 4 hr. Total lysates were analyzed by western blotting for the indicated proteins.

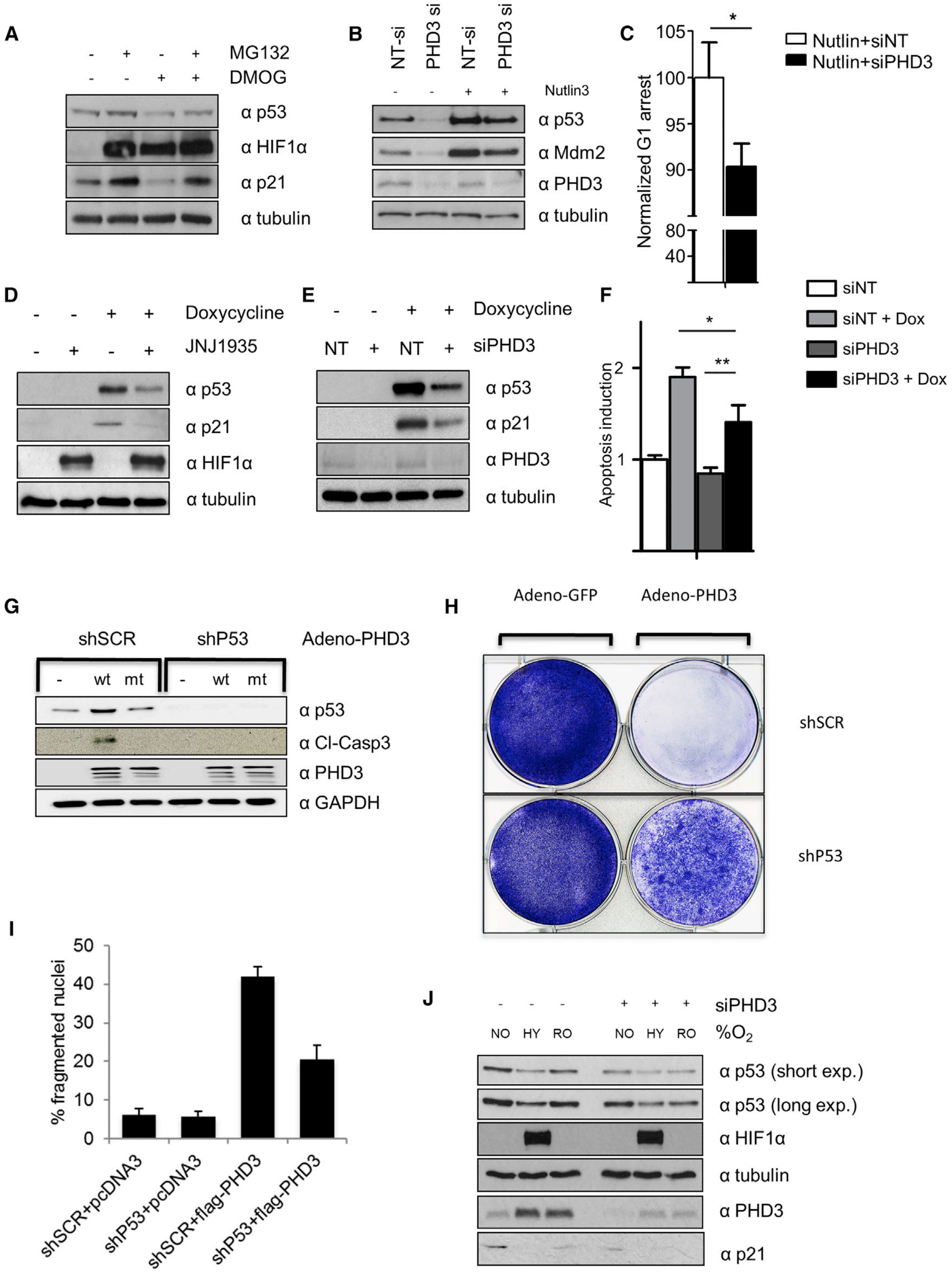

Figure 6. PHD3 Regulates p53 Levels and Downstream Signaling

(A) HepG2 cells were treated with DMSO, DMOG, or MG132 for 4 hr. Total lysates were analyzed by western blot with the indicated antibodies.

(B) HepG2 cells were transfected with non-target or PHD3 siRNA. After 48 hr, cells were treated with Nutlin-3. Total lysates were analyzed by western blot with the indicated antibodies.

(C) HepG2 cells were transfected with non-target or PHD3 siRNA. After 48 hr, cells were treated for 4 hr with Nutlin-3. Bar graphs represent the G1 arrest related to the Nutlin-3 treatment. Error bars are SEM, and n = 3.

(legend continued on next page)

cell-biological assays. S.L. performed apoptosis assays in U87 cells. A.Q. and A.K. performed RT-PCRs and cell line generation. N.R. generated mutants. K.W. assisted with mass spectrometry. J.R., J.C.A., S.S., and A.v.K. wrote and edited the manuscript.

DECLARATION OF INTERESTS

The authors declare no competing interests.

Received: September 4, 2017
Revised: March 30, 2018
Accepted: June 27, 2018
Published: July 31, 2018

REFERENCES

Alarcón, R., Koumenis, C., Geyer, R.K., Maki, C.G., and Giaccia, A.J. (1999). Hypoxia induces p53 accumulation through MDM2 down-regulation and inhibition of E6-mediated degradation. Cancer Res. *59*, 6046–6051.

Barrett, T.D., Palomino, H.L., Brondstetter, T.I., Kanelakis, K.C., Wu, X., Haug, P.V., Yan, W., Young, A., Hua, H., Hart, J.C., et al. (2011). Pharmacological characterization of 1-(5-chloro-6-(trifluoromethoxy)-1*H*-benzoimidazol-2-yl)-1*H*-pyrazole-4-carboxylic acid (JNJ-42041935), a potent and selective hypoxia-inducible factor prolyl hydroxylase inhibitor. Mol. Pharmacol. *79*, 910–920.

Bode, A.M., and Dong, Z. (2004). Post-translational modification of p53 in tumorigenesis. Nat. Rev. Cancer *4*, 793–805.

Bruick, R.K., and McKnight, S.L. (2001). A conserved family of prolyl-4-hydroxylases that modify HIF. Science *294*, 1337–1340.

Chen, B., Longtine, M.S., Sadovsky, Y., and Nelson, D.M. (2010). Hypoxia downregulates p53 but induces apoptosis and enhances expression of BAD in cultures of human syncytiotrophoblasts. Am. J. Physiol. Cell Physiol. *299*, C968–C976.

Cockman, M.E., Webb, J.D., Kramer, H.B., Kessler, B.M., and Ratcliffe, P.J. (2009). Proteomics-based identification of novel factor inhibiting hypoxia-inducible factor (FIH) substrates indicates widespread asparaginyl hydroxylation of ankyrin repeat domain-containing proteins. Mol. Cell. Proteomics *8*, 535–546.

Deschoemaeker, S., Di Conza, G., Lilla, S., Martín-Pérez, R., Mennerich, D., Boon, L., Hendrikx, S., Maddocks, O.D., Marx, C., Radhakrishnan, P., et al. (2015a). PHD1 regulates p53-mediated colorectal cancer chemoresistance. EMBO Mol. Med. *7*, 1350–1365.

Deschoemaeker, S., Di Conza, G., Lilla, S., Martin-Perez, R., Mennerich, D., Boon, L., Hendrikx, S., Maddocks, O.D., Marx, C., Radhakrishnan, P., et al. (2015b). PHD1 regulates p53-mediated colorectal cancer chemoresistance. EMBO Mol. Med. *7*, 1350–1365.

el-Deiry, W.S., Tokino, T., Velculescu, V.E., Levy, D.B., Parsons, R., Trent, J.M., Lin, D., Mercer, W.E., Kinzler, K.W., and Vogelstein, B. (1993). WAF1, a potential mediator of p53 tumor suppression. Cell *75*, 817–825.

el-Deiry, W.S., Harper, J.W., O'Connor, P.M., Velculescu, V.E., Canman, C.E., Jackman, J., Pietenpol, J.A., Burrell, M., Hill, D.E., Wang, Y., et al. (1994). WAF1/CIP1 is induced in p53-mediated G1 arrest and apoptosis. Cancer Res. *54*, 1169–1174.

Epstein, A.C., Gleadle, J.M., McNeill, L.A., Hewitson, K.S., O'Rourke, J., Mole, D.R., Mukherji, M., Metzen, E., Wilson, M.I., Dhanda, A., et al. (2001). *C. elegans* EGL-9 and mammalian homologs define a family of dioxygenases that regulate HIF by prolyl hydroxylation. Cell *107*, 43–54.

German, N.J., Yoon, H., Yusuf, R.Z., Murphy, J.P., Finley, L.W., Laurent, G., Haas, W., Satterstrom, F.K., Guarnerio, J., Zaganjor, E., et al. (2016). PHD3 loss in cancer enables metabolic reliance on fatty acid oxidation via deactivation of ACC2. Mol. Cell *63*, 1006–1020.

Gogna, R., Madan, E., Kuppusamy, P., and Pati, U. (2012). Re-oxygenation causes hypoxic tumor regression through restoration of p53 wild-type conformation and post-translational modifications. Cell Death Dis. *3*, e286.

Graeber, T.G., Peterson, J.F., Tsai, M., Monica, K., Fornace, A.J., Jr., and Giaccia, A.J. (1994). Hypoxia induces accumulation of p53 protein, but activation of a G1-phase checkpoint by low-oxygen conditions is independent of p53 status. Mol. Cell. Biol. *14*, 6264–6277.

Grand, R.J., Turnell, A.S., Mason, G.G., Wang, W., Milner, A.E., Mymryk, J.S., Rookes, S.M., Rivett, A.J., and Gallimore, P.H. (1999). Adenovirus early region 1A protein binds to mammalian SUG1-a regulatory component of the proteasome. Oncogene *18*, 449–458.

Hammond, E.M., and Giaccia, A.J. (2005). The role of p53 in hypoxia-induced apoptosis. Biochem. Biophys. Res. Commun. *331*, 718–725.

Hansson, L.O., Friedler, A., Freund, S., Rudiger, S., and Fersht, A.R. (2002). Two sequence motifs from HIF-1alpha bind to the DNA-binding site of p53. Proc. Natl. Acad. Sci. USA *99*, 10305–10309.

Henze, A.T., Garvalov, B.K., Seidel, S., Cuesta, A.M., Ritter, M., Filatova, A., Foss, F., Dopeso, H., Essmann, C.L., Maxwell, P.H., et al. (2014). Loss of PHD3 allows tumours to overcome hypoxic growth inhibition and sustain proliferation through EGFR. Nat. Commun. *5*, 5582.

Hewitson, K.S., McNeill, L.A., Riordan, M.V., Tian, Y.M., Bullock, A.N., Welford, R.W., Elkins, J.M., Oldham, N.J., Bhattacharya, S., Gleadle, J.M., et al. (2002). Hypoxia-inducible factor (HIF) asparagine hydroxylase is identical to factor inhibiting HIF (FIH) and is related to the cupin structural family. J. Biol. Chem. *277*, 26351–26355.

Högel, H., Rantanen, K., Jokilehto, T., Grenman, R., and Jaakkola, P.M. (2011). Prolyl hydroxylase PHD3 enhances the hypoxic survival and G1 to S transition of carcinoma cells. PLoS One *6*, e27112.

Hoppe-Seyler, F., and Butz, K. (1993). Repression of endogenous p53 transactivation function in HeLa cervical carcinoma cells by human papillomavirus type 16 E6, human mdm-2, and mutant p53. J. Virol. *67*, 3111–3117.

(D) p53 null Saos-2 were treated with DOX (1.25 μg/mL) for 24 hr, and after this time JNJ or DMSO was added for 4 hr. Proteins of the total lysates were separated by PAGE, electroblotted, and detected by the indicated antibodies.
(E) p53 null Saos-2 were transfected with either siNT or siPHD3 for 24 hr prior to treatment with DOX (1.25 μg/mL) for 24 hr. Total lysates were tested by western blot for the indicated proteins.
(F) p53 null Saos-2 were transfected with either siNT or siPHD3 for 24 hr prior to 32-hr treatment with DOX (1.25 μg/mL). Bar graphs represent normalization of number of apoptotic cells measured by YoPro staining. Error bars are SEM, and n = 3.
(G) U87 cells were stably infected with scrambled or p53-specific shRNA constructs. These cells were transiently infected with an adenovirus driving exogenous wt or inactive mutant PHD3 expression or a GFP control. Total lysates were analyzed 48 hr post-infection by western blot with the indicated antibodies.
(H) U87 cells stably infected with scrambled or p53-specific shRNA constructs were infected with an adenovirus driving exogenous PHD3 expression or a GFP control. Cells were seeded and stained with crystal violet 2 weeks post-transduction.
(I) U87 cells stably infected with scrambled or p53-specific shRNA constructs were transiently transduced with plasmids expressing exogenous Flag-PHD3 expression or a Flag tag control vector. Bar graphs represent the percentage of U87 exhibiting apoptotic changes, visualized with Hoechst staining for living cells (only Flag-positive cells were scored). Error bars are SD, and n = 3.
(J) HepG2 cells were transfected with non-target or PHD3 siRNA. After 24 hr, cells were cultured in 1% oxygen (HY) or in normoxia (NO). One-half of the hypoxic cells were taken out of hypoxia and culture for 60 min in normoxia (RO). Total lysates were analyzed by western blot with the indicated antibodies.
Two-tailed, equal distribution Student's t test was employed to test for statistical difference with values of $^{*}p < 0.05$ and $^{**}p < 0.01$.

Ivan, M., Kondo, K., Yang, H., Kim, W., Valiando, J., Ohh, M., Salic, A., Asara, J.M., Lane, W.S., and Kaelin, W.G., Jr. (2001). HIFalpha targeted for VHL-mediated destruction by proline hydroxylation: implications for O2 sensing. Science *292*, 464–468.

Jaakkola, P., Mole, D.R., Tian, Y.M., Wilson, M.I., Gielbert, J., Gaskell, S.J., von Kriegsheim, A., Hebestreit, H.F., Mukherji, M., Schofield, C.J., et al. (2001). Targeting of HIF-alpha to the von Hippel-Lindau ubiquitylation complex by O_2-regulated prolyl hydroxylation. Science *292*, 468–472.

Janke, K., Brockmeier, U., Kuhlmann, K., Eisenacher, M., Nolde, J., Meyer, H.E., Mairbäurl, H., and Metzen, E. (2013). Factor inhibiting HIF-1 (FIH-1) modulates protein interactions of apoptosis-stimulating p53 binding protein 2 (ASPP2). J. Cell Sci. *126*, 2629–2640.

Koumenis, C., Alarcon, R., Hammond, E., Sutphin, P., Hoffman, W., Murphy, M., Derr, J., Taya, Y., Lowe, S.W., Kastan, M., and Giaccia, A. (2001). Regulation of p53 by hypoxia: dissociation of transcriptional repression and apoptosis from p53-dependent transactivation. Mol. Cell. Biol. *21*, 1297–1310.

Lee, D.H., and Goldberg, A.L. (1998). Proteasome inhibitors: valuable new tools for cell biologists. Trends Cell Biol. *8*, 397–403.

Lee, S., Nakamura, E., Yang, H., Wei, W., Linggi, M.S., Sajan, M.P., Farese, R.V., Freeman, R.S., Carter, B.D., Kaelin, W.G., Jr., and Schlisio, S. (2005). Neuronal apoptosis linked to EglN3 prolyl hydroxylase and familial pheochromocytoma genes: developmental culling and cancer. Cancer Cell *8*, 155–167.

Lee, S.B., Frattini, V., Bansal, M., Castano, A.M., Sherman, D., Hutchinson, K., Bruce, J.N., Califano, A., Liu, G., Cardozo, T., et al. (2016). An ID2-dependent mechanism for VHL inactivation in cancer. Nature *529*, 172–177.

Levine, A.J. (1997). p53, the cellular gatekeeper for growth and division. Cell *88*, 323–331.

Li, M., Chen, D., Shiloh, A., Luo, J., Nikolaev, A.Y., Qin, J., and Gu, W. (2002). Deubiquitination of p53 by HAUSP is an important pathway for p53 stabilization. Nature *416*, 648–653.

Li, T., Kon, N., Jiang, L., Tan, M., Ludwig, T., Zhao, Y., Baer, R., and Gu, W. (2012). Tumor suppression in the absence of p53-mediated cell-cycle arrest, apoptosis, and senescence. Cell *149*, 1269–1283.

Lowe, S.W., and Ruley, H.E. (1993). Stabilization of the p53 tumor suppressor is induced by adenovirus 5 E1A and accompanies apoptosis. Genes Dev. *7*, 535–545.

Lu, W., Pochampally, R., Chen, L., Traidej, M., Wang, Y., and Chen, J. (2000). Nuclear exclusion of p53 in a subset of tumors requires MDM2 function. Oncogene *19*, 232–240.

Ma, J., Martin, J.D., Xue, Y., Lor, L.A., Kennedy-Wilson, K.M., Sinnamon, R.H., Ho, T.F., Zhang, G., Schwartz, B., Tummino, P.J., and Lai, Z. (2010). C-terminal region of USP7/HAUSP is critical for deubiquitination activity and contains a second mdm2/p53 binding site. Arch. Biochem. Biophys. *503*, 207–212.

Michael, D., and Oren, M. (2003). The p53-Mdm2 module and the ubiquitin system. Semin. Cancer Biol. *13*, 49–58.

Nakano, K., Bálint, E., Ashcroft, M., and Vousden, K.H. (2000). A ribonucleotide reductase gene is a transcriptional target of p53 and p73. Oncogene *19*, 4283–4289.

Obrig, T.G., Culp, W.J., McKeehan, W.L., and Hardesty, B. (1971). The mechanism by which cycloheximide and related glutarimide antibiotics inhibit peptide synthesis on reticulocyte ribosomes. J. Biol. Chem. *246*, 174–181.

Pan, Y., Oprysko, P.R., Asham, A.M., Koch, C.J., and Simon, M.C. (2004). p53 cannot be induced by hypoxia alone but responds to the hypoxic microenvironment. Oncogene *23*, 4975–4983.

Rantanen, K., Pursiheimo, J., Högel, H., Himanen, V., Metzen, E., and Jaakkola, P.M. (2008). Prolyl hydroxylase PHD3 activates oxygen-dependent protein aggregation. Mol. Biol. Cell *19*, 2231–2240.

Rodriguez, J., Pilkington, R., Garcia Munoz, A., Nguyen, L.K., Rauch, N., Kennedy, S., Monsefi, N., Herrero, A., Taylor, C.T., and von Kriegsheim, A. (2016). Substrate-trapped interactors of PHD3 and FIH cluster in distinct signaling pathways. Cell Rep. *14*, 2745–2760.

Schlisio, S., Kenchappa, R.S., Vredeveld, L.C., George, R.E., Stewart, R., Greulich, H., Shahriari, K., Nguyen, N.V., Pigny, P., Dahia, P.L., et al. (2008). The kinesin KIF1Bbeta acts downstream from EglN3 to induce apoptosis and is a potential 1p36 tumor suppressor. Genes Dev. *22*, 884–893.

Sciorra, V.A., Sanchez, M.A., Kunibe, A., and Wurmser, A.E. (2012). Suppression of glioma progression by Egln3. PLoS One *7*, e40053.

Sermeus, A., Rebucci, M., Fransolet, M., Flamant, L., Desmet, D., Delaive, E., Arnould, T., and Michiels, C. (2013). Differential effect of hypoxia on etoposide-induced DNA damage response and p53 regulation in different cell types. J. Cell. Physiol. *228*, 2365–2376.

Sheikh, M.S., Li, X.S., Chen, J.C., Shao, Z.M., Ordonez, J.V., and Fontana, J.A. (1994). Mechanisms of regulation of WAF1/Cip1 gene expression in human breast carcinoma: role of p53-dependent and independent signal transduction pathways. Oncogene *9*, 3407–3415.

Sheng, Y., Saridakis, V., Sarkari, F., Duan, S., Wu, T., Arrowsmith, C.H., and Frappier, L. (2006). Molecular recognition of p53 and MDM2 by USP7/HAUSP. Nat. Struct. Mol. Biol. *13*, 285–291.

Talis, A.L., Huibregtse, J.M., and Howley, P.M. (1998). The role of E6AP in the regulation of p53 protein levels in human papillomavirus (HPV)-positive and HPV-negative cells. J. Biol. Chem. *273*, 6439–6445.

Tate, S., Larsen, B., Bonner, R., and Gingras, A.C. (2013). Label-free quantitative proteomics trends for protein-protein interactions. J. Proteomics *81*, 91–101.

Turriziani, B., Garcia-Munoz, A., Pilkington, R., Raso, C., Kolch, W., and von Kriegsheim, A. (2014). On-beads digestion in conjunction with data-dependent mass spectrometry: a shortcut to quantitative and dynamic interaction proteomics. Biology (Basel) *3*, 320–332.

Ullah, K., Rosendahl, A.H., Izzi, V., Bergmann, U., Pihlajaniemi, T., Mäki, J.M., and Myllyharju, J. (2017). Hypoxia-inducible factor prolyl-4-hydroxylase-1 is a convergent point in the reciprocal negative regulation of NF-κB and p53 signaling pathways. Sci. Rep. *7*, 17220.

Vassilev, L.T. (2004). Small-molecule antagonists of p53-MDM2 binding: research tools and potential therapeutics. Cell Cycle *3*, 419–421.

Wang, F., He, L., Huangyang, P., Liang, J., Si, W., Yan, R., Han, X., Liu, S., Gui, B., Li, W., et al. (2014). JMJD6 promotes colon carcinogenesis through negative regulation of p53 by hydroxylation. PLoS Biol. *12*, e1001819.

Warboys, C.M., de Luca, A., Amini, N., Luong, L., Duckles, H., Hsiao, S., White, A., Biswas, S., Khamis, R., Chong, C.K., et al. (2014). Disturbed flow promotes endothelial senescence via a p53-dependent pathway. Arterioscler. Thromb. Vasc. Biol. *34*, 985–995.

Weinmann, M., Jendrossek, V., Güner, D., Goecke, B., and Belka, C. (2004). Cyclic exposure to hypoxia and reoxygenation selects for tumor cells with defects in mitochondrial apoptotic pathways. FASEB J. *18*, 1906–1908.

Xie, L., Pi, X., Mishra, A., Fong, G., Peng, J., and Patterson, C. (2012). PHD3-dependent hydroxylation of HCLK2 promotes the DNA damage response. J. Clin. Invest. *122*, 2827–2836.

Yuan, J., Luo, K., Zhang, L., Cheville, J.C., and Lou, Z. (2010). USP10 regulates p53 localization and stability by deubiquitinating p53. Cell *140*, 384–396.

Cell Reports, Volume *24*

Supplemental Information

PHD3 Regulates p53 Protein Stability

by Hydroxylating Proline 359

Javier Rodriguez, Ana Herrero, Shuijie Li, Nora Rauch, Andrea Quintanilla, Kieran Wynne, Aleksandar Krstic, Juan Carlos Acosta, Cormac Taylor, Susanne Schlisio, and Alex von Kriegsheim

Figure S1

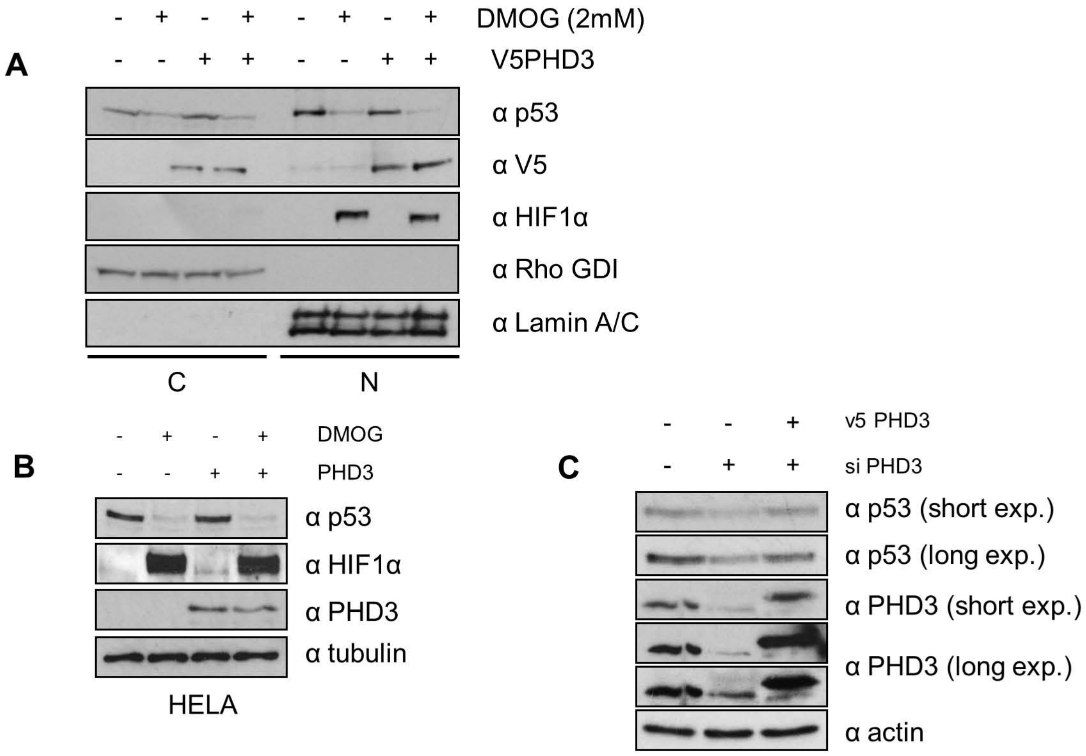

Figure S1 PHD3 regulates p53 protein stability. Related to Figure 2. (A) HeLa cells were transfected with an empty vector control and/or V5-tagged PHD3. 24 hours post-transfection the cells were treated or not with DMOG for 4 hours. Cells were lysed and the nuclear or cytoplasmic compartment were fractionated. Proteins were separated by PAGE, electroblotted and detected by the indicated antibodies. (B) HeLa cells were transfected with an empty vector control and/or V5-tagged PHD3. 24 hours post-transfection the cells were treated or not with DMOG for 4 hours. Cells were lysed and proteins were separated by PAGE, electroblotted and detected by the indicated antibodies.(C) HeLa cells were transfected with PHD3 specific siRNA. 24 hours post-transfection the cells were transfected with V5-PHD3 or not. Cells were lysed and proteins were separated by PAGE, electroblotted and detected by the indicated antibodies.

Figure S2

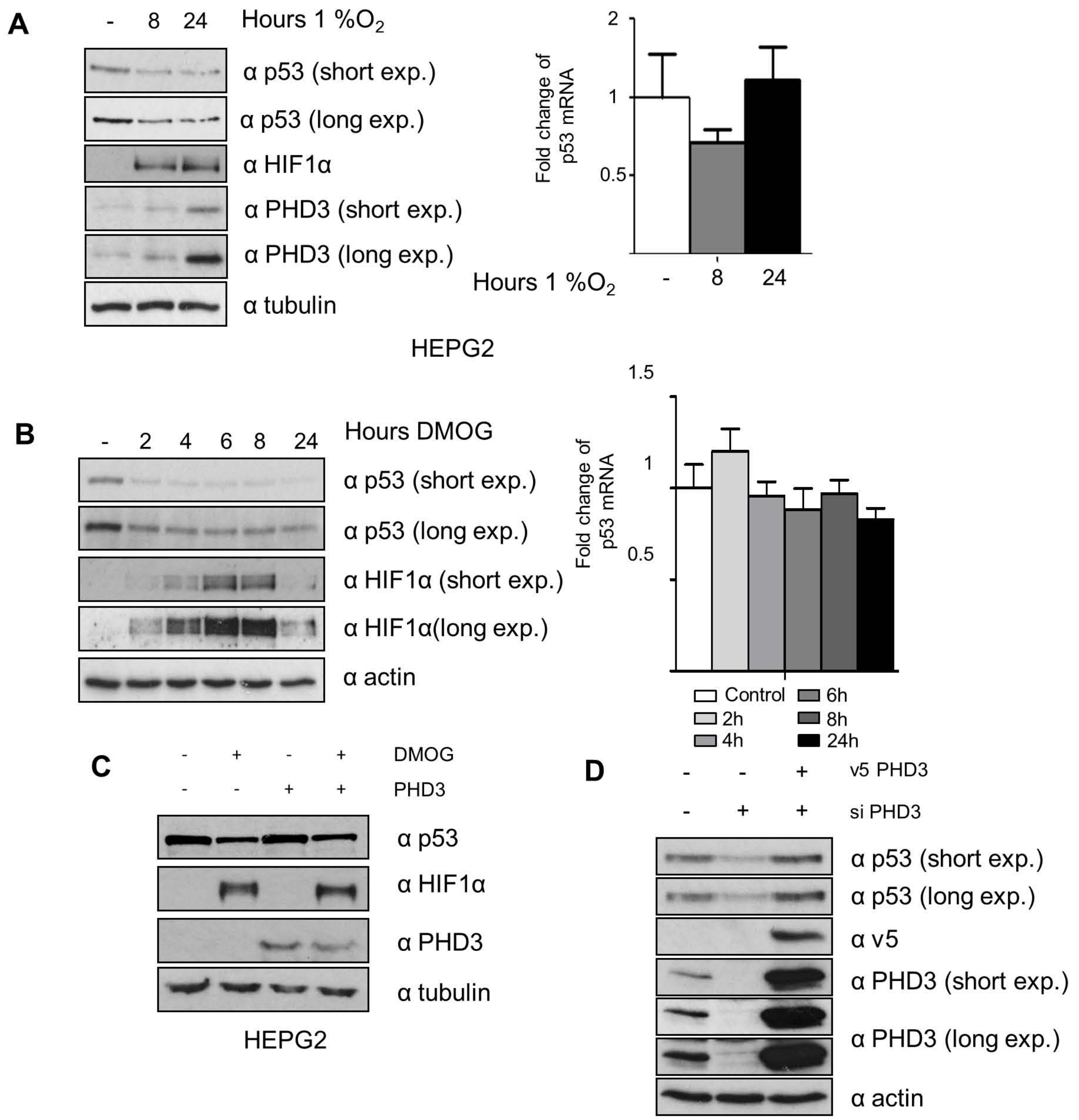

Figure S2 PHD3 regulates p53 protein stability and ubiquitination. Related to Figure 3. (A) HepG2 cells were cultured in 1% oxygen as indicated, lysed and proteins were separated by PAGE, electroblotted and detected by the indicated antibodies. In parallel, mRNA was extracted and quantified by RT-PCR (second panel) (B) HepG2 cells were treated with DMOG as indicated lysed and proteins were separated by PAGE, electroblotted and detected by the indicated antibodies. In parallel, mRNA was extracted and quantified by RT-PCR (second panel). (C) HepG2 cells were transfected with an empty vector control and/or V5-tagged PHD3. 24 hours post-transfection the cells were treated or not with DMOG for 4 hours. Cells were lysed and proteins were separated by PAGE, electroblotted and detected by the indicated antibodies. .(D) HepG2 cells were transfected with PHD3 specific siRNA. 24 hours post-transfection the cells were transfected with V5-PHD3 or not. Cells were lysed and proteins were separated by PAGE, electroblotted and detected by the indicated antibodies.

Figure S3

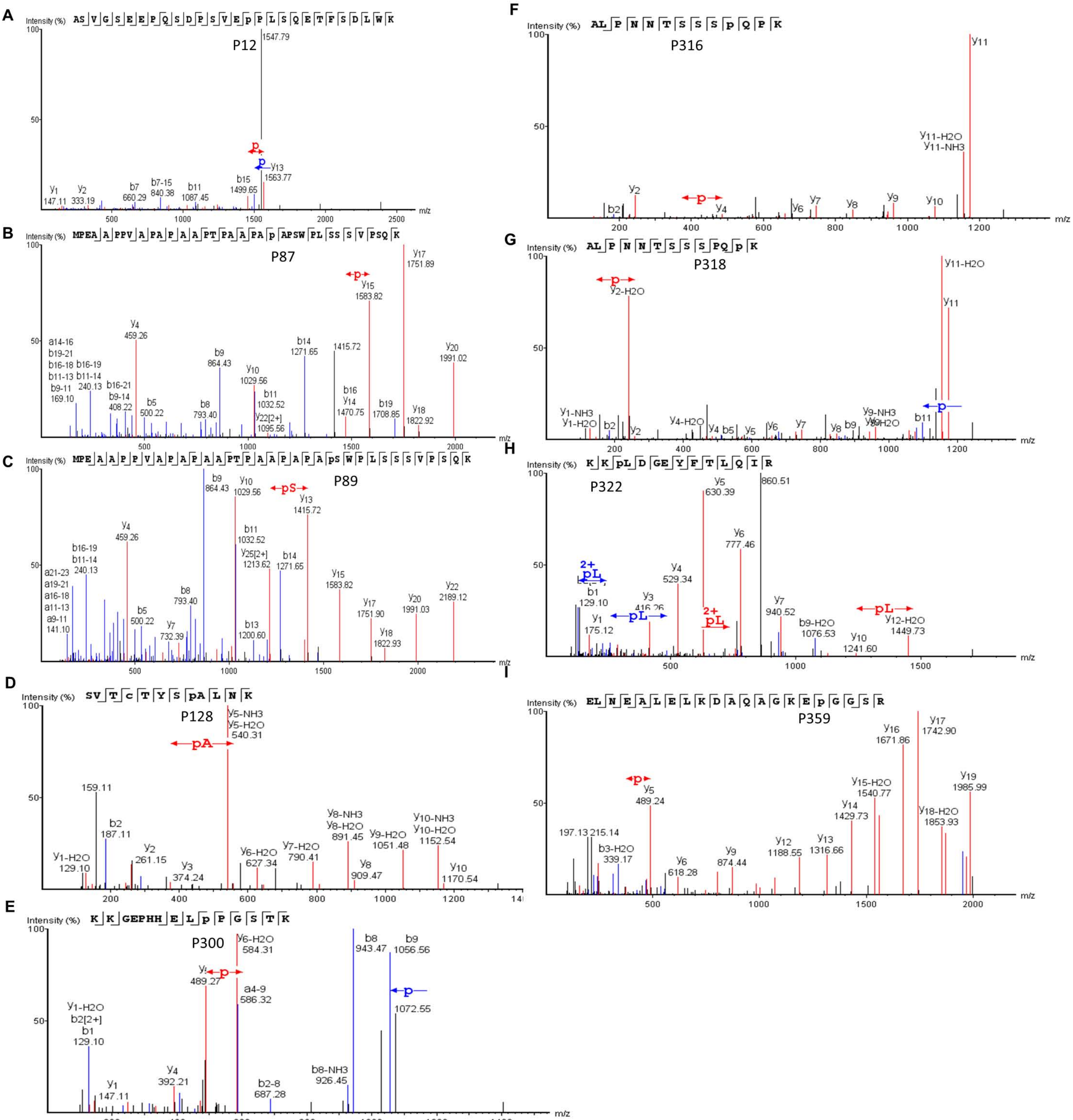
A
ASVGSEEPQSDPSVEpPLSQETFSDLWK
P12
B
MPEAAPPVAPAPAAPTPAAPAPAPSWPLSSSVPSQK
P87
C
MPEAAPPVAPAPAAPTPAAPAPApSWPLSSSVPSQK
P89
D
SVTCTYSpALNK
P128
E
KKGEPHHELpPGSTK
P300
F
ALPNNTSSSpQPK
P316
G
ALPNNTSSSPQpK
P318
H
KKpLDGEYFTLQIR
P322
I
ELNEALELKDAQAGKEpGGSR
P359

Figure S3. Fragmentation spectra of proline hydroxylated peptides detected in following an *in vitro* hydroxylation assay with GSTp53 as substrate. Related to Figure 4.

(A) ASVGSEEPQSDPSVEP(ox)PLSQETFSDLWF,

(B) MPEAAPPVAPAPAAPTPAAPAP(ox)APSWPLSSSVPSQK**,**

(C) MPEAAPPVAPAPAAPTPAAPAP(ox)AP(ox)SWPLSSSVPSQK

(D) SVTCTYSP(ox)ALNK

(E) KKGEPHHELP(ox)PGSTK

(F) ALPNNTSSSP(ox)QPK

(G) ALPNNTSSSPQP(ox)K

(H) KKP(ox)LDGEYFTLQIR

(I)ELNEALELKDAQAGKEP(ox)GGSR

Figure S4

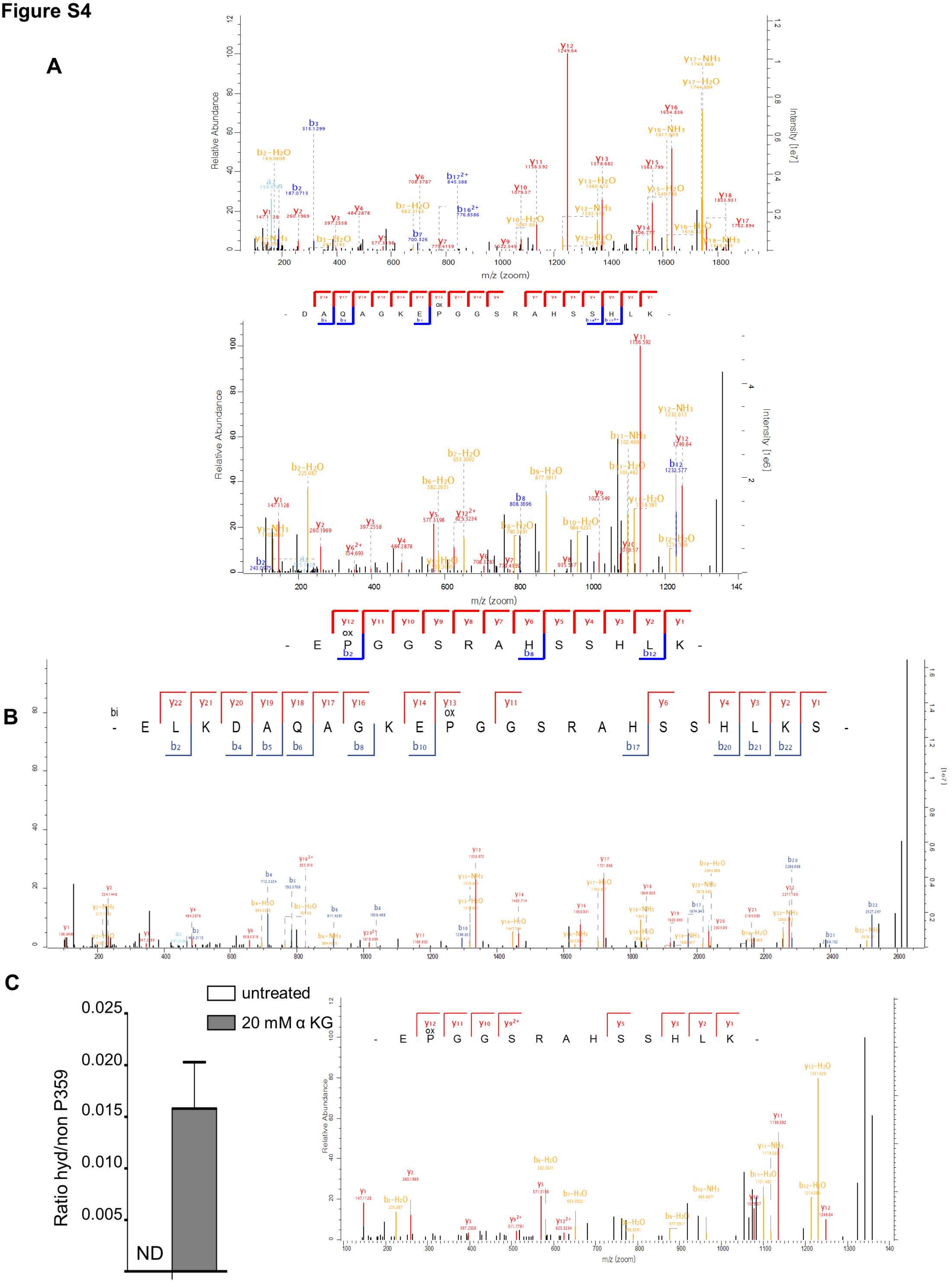
A
Relative Abundance
Intensity [1e7]
m/z (zoom)
- D A Q A G K E P G G S R A H S S H L K -
ox
Intensity [1e6]
- E P G G S R A H S S H L K -
B
bi
- E L K D A Q A G K E P G G S R A H S S H L K S -
[1e7]
C
untreated
20 mM α KG
Ratio hyd/non P359
0.025
0.020
0.015
0.010
0.005
ND

Figure S4. Representative fragmentation spectra of the oxidation of p53 at the P359. Related to Figure 4.

(A) HCD fragmentation spectra of the peptide EP(ox)GGSRAHSSHLK and DAQAGKEP(ox)GGSRAHSSHdetected in a FLAG p53. (B) Fragmentation spectra of the biotinylated peptide ELKDAQAGKEP(ox)GGSRAHSSHLK. (C) Ratio of hydroxylated/non-hydroxylated peptide following an in vitro hydroxylation reaction with 20 mM alpha-ketoglutarate and the untreated peptide. Fragmentation spectra of EP(ox)GGSRAHSSHLK derived from the trypsin digested, biotinylated peptide ELKDAQAGKEPGGSRAHSSHLKS hydroxylated in vitro.

Figure S5

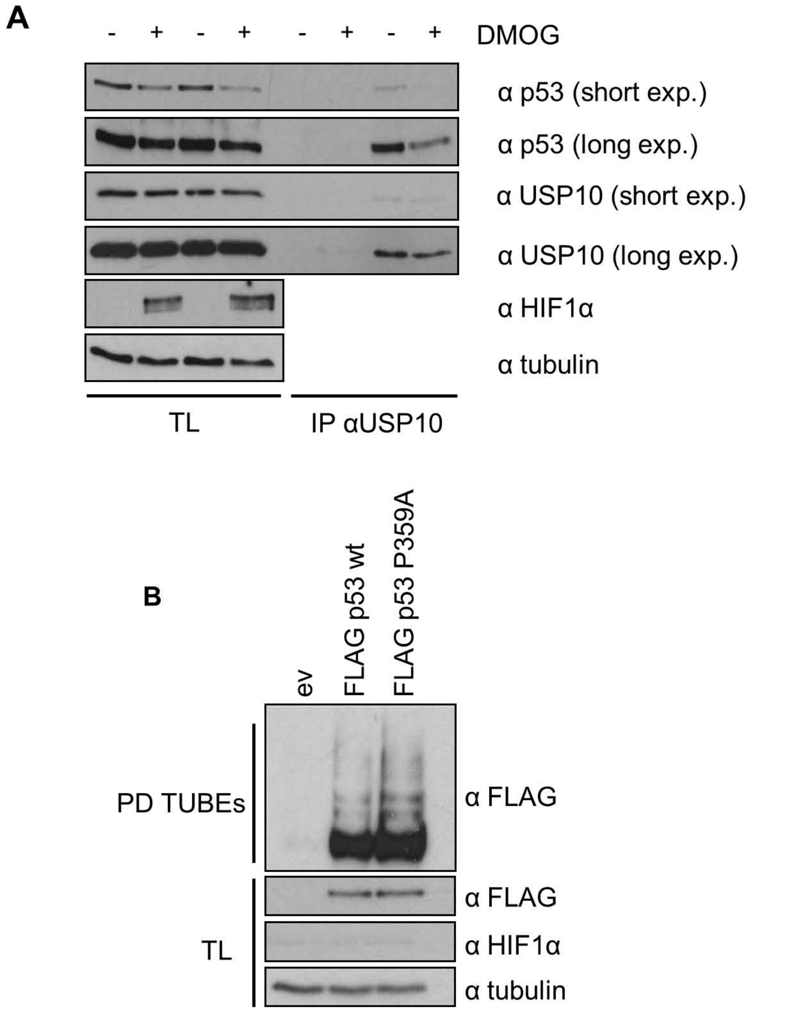

Figure S5. P53 and USP10 interact endogenously. Related to Figure 5.
(A) HepG2 cells were treated with or without DMOG for 4 hours. The cells were lysed and endogenous USP10 was immunoprecipitated. Total lysates and the corresponding immunoprecipitates were probed for the indicated proteins. (B) HepG2 cells were transfected with Flag-p53 wt and Flag-p53 P359A. Cells were lysed and ubiquitinated proteins were precipitated with TUBE-agarose (PD). Proteins were separated by PAGE and electroblotted. Ubiquitinated Flag-p53 was detected by an anti-Flag antibody (PD) and changes in expression in total lysate were blotted separately (TL).

Figure S6

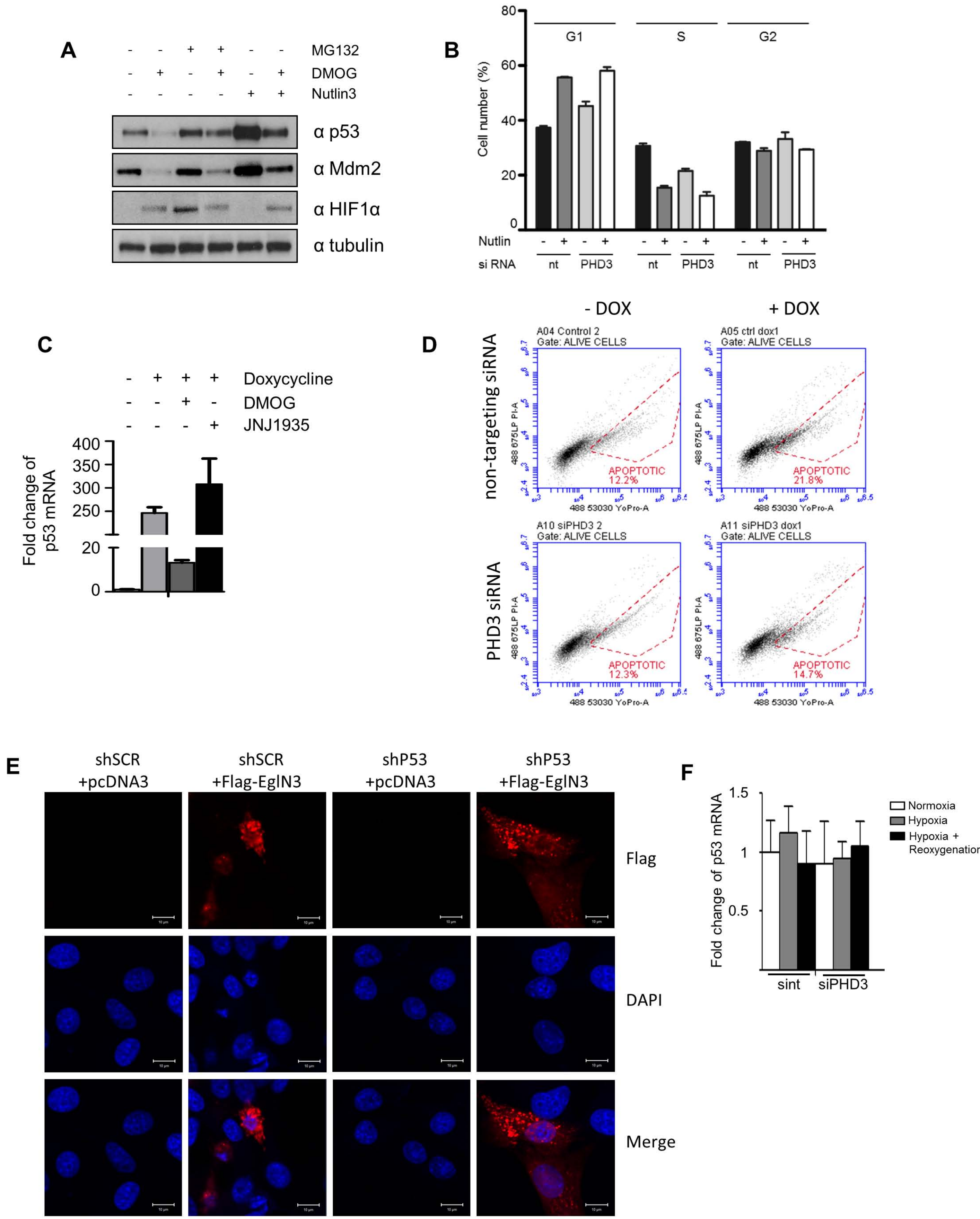
A
MG132
DMOG
Nutlin3
α p53
α Mdm2
α HIF1α
α tubulin
B
G1
S
G2
Cell number (%)
80
60
40
20
0
Nutlin
si RNA
nt
PHD3
C
Doxycycline
DMOG
JNJ1935
Fold change of p53 mRNA
400
350
300
250
20
0
D
- DOX
+ DOX
non-targeting siRNA
PHD3 siRNA
A04 Control 2
Gate: ALIVE CELLS
A05 ctrl dox1
Gate: ALIVE CELLS
A10 siPHD3 2
Gate: ALIVE CELLS
A11 siPHD3 dox1
Gate: ALIVE CELLS
APOPTOTIC
12.2%
21.8%
12.3%
14.7%
488 675LP PI-A
488 53030 YoPro-A
E
shSCR +pcDNA3
shSCR +Flag-EglN3
shP53 +pcDNA3
shP53 +Flag-EglN3
Flag
DAPI
Merge
F
Fold change of p53 mRNA
1.5
1
0.5
Normoxia
Hypoxia
Hypoxia + Reoxygenation
sint
siPHD3

Figure S6. PHD3 regulates p53 levels and downstream signalling. Related to Figure 6.
(A) HepG2 cells were treated with DMSO, DMOG or MG132 for four hours. Total lysates were analysed by Western blot with the indicated antibodies. (B) HepG2 cells were transfected with non-target or PHD3 siRNA. After 48 hours cells were treated for 4 hours with Nutlin3. Bar graphs represent the % of cells in G1, G2 and S. Error bars are SEM n=3. (C) p53-null Saos-2 were treated with doxycycline (1.25 µg/ml) for 24 hours. After this time these cells were treated with the combination of hydroxylases inhibitors that is showed at the panel. Cells were harvested for quantitative RT-PCR assays. The expression level of p53 was normalized to endogenous actin mRNA levels. The values plotted are means, SD of N=3 independent experiments for each condition. (D) p53-null Saos-2 were transfected with either siNT or siPHD3 for 24 h prior to 32 h treatment with Doxycycline (1.25 µg/ml). Representative graphs showing gating for apoptotic cells measured by YoPro/propidum iodide co-staining to detect apoptotic cells. (E) U87 cells stably infected with scrambled or p53-specific shRNA constructs were transiently transduced with plasmids expressing exogenous Flag-PHD3 expression or a control vector. Cells were stained with anti-Flag antibody or DAPI for nuclei. (F) HepG2 cells were transfected with non-target or PHD3 siRNA. After 24 hours cells were cultured in 1% oxygen (HY) or in normoxia (NO). Half of the hypoxic cells were taken out of hypoxia and culture for 60 minutes in normoxia (RO). The expression level of p53 was normalized to endogenous actin mRNA levels. The values plotted are means SD of N=3 independent

Supplemental Experimental Procedures

Cell culture: HEK293T, U87 and p53-inducible human tumour Saos-2 cells were cultured in Dulbecco's modified Eagle medium (DMEM) supplemented with 2 mM glutamine (Invitrogen) and 10% foetal calf serum (Invitrogen). HepG2 were cultured in the same conditions, but using the DMEM low glucose (1 g/L). Plasmids and siRNA oligonucleotides were transfected with Lipofectamine 2000 (Invitrogen) according to the vendor's instructions. P53-inducible Saos-2 cells were a kind gift from Dr Bob Ludwig (Prof. Vousden's group).

Materials: All antibodies were from commercial sources: anti-FLAG M2 peroxidase was obtained from Sigma Aldrich (F1804), anti-HIF1α was from BD Biosciences (610958), anti-p53, anti-tubulin, anti-GST, anti-HA, anti-His, anti-GST and anti-p21 were purchased from Santa Cruz(sc-126/sc-8035/sc-138/sc-7392/sc-803/sc-138/sc-6246, 1:1,000 dilution), anti-PHD3 was from Novus Biologicals (NB100-139), anti-USP10, anti-cleaved caspase 3 (Asp175) and anti-p53 was purchased from Cell Signalling Technologies (5553, 9664, 9282), and anti-V5 was obtained from Invitrogen (R96025). DMOG was obtained from Cayman Chemical (71210), MG132 was purchased from Sigma (M7449), the PHD-specific inhibitor JNJ-42041935 was purchased from Merck Millipore (400093), Nutlin3 was from Sigma (N6287), and cycloheximide was from Sigma (C4859/M7449).

Plasmids and siRNAs: HA-PHD3 and HA-PHD3 H196A were purchased from Addgene (18960/22717), and PHD3 H135A/D137A was a gift from Prof. Semenza Flag-USP7 and Flag-USP10 were from Addgene (16655/22543). FLAG-His-p53 was a gift from Cynthia Coffill. Flag–His-p53-P359A mutant was made using the QuikChange kit (Stratagene) using following oligos
CTAGGTATATGGACTTAAAAGCCTTGGGTTGTGGAGGCAATG (forward)
CATTGCCTCCACAACCCAAGGCTTTTAAGTCCATATACCTAG (reverse). The non-targeting siRNA (siNT), the siRNA targeting PHD3 (siPHD3) and the siRNA targeting USP10 were purchased from Dharmacon (ONTARGETplusSMARTpool).

Cell lysis and immunoprecipitation: Cells were lysed in ice-cold lysis buffer (1% Triton-x100, 20 mM Tris-HCl (pH 7.5), 150 mM NaCl), supplemented with protease (5 μg/ml leupeptin, 2,2 μg/ml aprotinin) and phosphatase inhibitors (20 mM β-glycerophosphate). Lysates were cleared of debris by centrifugation at 20,000 x g for 10 min in a benchtop centrifuge (4ºC). For immunoprecipitation anti-Flag-M2 beads (Sigma Aldrich) or anti-V5 beads (Sigma-Aldrich) were added to the cleared lysates and incubated at 4ºC under end-to-end rotation for 2 hours. Beads were washed three times with PBS 1X-1% NP40 and boiled off in Laemmli buffer.

Immunoblotting: Total lysates and affinity precipitates were fractionated by SDS-PAGE and transferred onto nitrocellulose filters. Immuno-complexes were visualized by enhanced chemiluminescence detection (GE Healthcare) with horseradish peroxidase–conjugated secondary antibodies (Bio-Rad Laboratories). Experiments were repeated at least 3 times.

Endogenous co-immunoprecipitation assays: Cells were washed in ice-cold PBS and lysed in 1% Triton-x100, 20 mM Tris-HCl (pH 7.5), 150 mM NaCl, supplemented with protease (5 μg/ml leupeptin, 2,2 μg/ml aprotinin) and phosphatase inhibitors (20 mM β-glycerophosphate). Lysates were cleared by centrifugation, 0.1 vol was separated to be loaded as the total lysate, and the rest were incubated with antibody rocking at 4°C for 1 h (a pre-immune antibody was used as negative control). Protein G–Sepharose was added and agitated at 4°C for 2 h. Beads were collected and washed three times with NP-40/PBS. Pellets were resuspended in 5× Laemmli buffer, boiled for 5 min, and fractionated by SDS-PAGE.

Mass spectrometry: HEK293T cells were transfected with either empty vector, a V5-tagged hydroxylases or Flag-tagged p53 and treated 24 hours post-transfection with either 2 mM DMOG or DMSO for 4 hours. The cells were lysed and we immunoprecipitated the protein with anti-V5 or anti FLAG agarose for 2 hour. The samples were digested with trypsin or Lys-C and processed as previously described (Turriziani et al., 2014). Desalted peptides were analysed on a Fusion Lumos mass spectrometer (Thermo, Germany).

In vitro hydroxylation assays:

Peptide hydroxylation: N-terminally biotinylated synthetic peptide ELKDAQAGKEPGGSRAHSSHLKS (Peptides & elephants, Germany) were incubated with streptavidin beads for 1 hour, after this incubation were washed 3 times with PBS. Finally were incubated with lysates derived from HEK293T cells transiently transfected with PHD3 wt or

PHD3 H196A as previously described. After the reaction was completed the peptides were eluted with biotin, desalted and analysed by LC-MS/MS.

p53 in vitro hydroxylation: The immobilized GST-p53 protein was incubated at 30°C for 1 h with HEK293T cell lysates supplemented with 100 μM FeCl2, 5 mM ascorbate and 1 mM α-ketoglutarate. After this incubation the beads were washed, digested with trypsin as described (Turriziani et al., 2014) and analysed by LC-MS/MS.

***In Vitro* Binding Assays:** GST fusion proteins were expressed in E. coli and purified. HEK293T cells transiently were transfected with V5-PHD3 and treated with 2 Mm DMOG or DMSO. Co-IP, GST pull-down, and immunoblot assays were performed as described (Rodriguez J et al., 2010).

Peptide pulldown assay: Peptides were purified with Streptavidin sepharose (Amersham Biosc.), and incubated with lysates of HEK293T cells that were transiently transfected with the indicated plasmids. GST pull-down, and immunoblot assays were performed as described (Rodriguez J et al., 2010).

Cell cycle analysis by PI staining: HepG2 cells were transfected with the specific siRNA for PDH3 (60pmol). After 48 hours, cells were treated with Nutlin-3 (10μM) for 5 hours prior to collection. After trypsinization (0.05% Trypsin-EDTA) and collection in growth medium (5ml), cells were washed once with ice-cold PBS, and finally re-suspended in 100 μl PBS. Addition of 900 μl of ice-cold ethanol (70%) was performed for cellular fixation with overnight incubation at 4 C. Prior Fluorescence-activated cell sorter (FACS) analysis, cells were re-suspended in 300 μl PBS containing Propidium Iodide (10 μg/ml) and RNAse A (100 μg/ml) and incubated in dark at room temperature for 30 minutes. Each sample was analysed, in duplicates, with BD Accuri C6, using FL-3 for PI (ex 488; em 670LP nm). Cells were gated by forward and side scatter in order to remove debris and doublets, resulting in the final “singlets in scatter” gating, and the DNA content per cell was measured using the BDAcuri software.

Apoptosis analysis by YO-PRO1/PI staining: p53-inducible Saos cells were transfected with siRNA for specific knockdown of PHD3 (60pmol) and treated with doxycycline (1.25 μg/ml) for 36 hours to induce p53 expression. Apoptosis levels were measured by Yo-Pro-1 uptake, which only permeates the membranes of apoptotic cells, and fluorescently labels the nucleus. Cells were collected by trypsinization (0.05% Trypsin-EDTA) in growth medium (5ml). Cells were washed with ice-cold PBS and incubated in PBS containing Yo-Pro-1 (Thermo Fisher Scientific) at 100 mM concentration. After 5 minutes Propidium Iodide at 10 μg/ml was added for another 5 minutes. Apoptotic levels were measured by assessing Fluorescence-activated cell sorter (FACS) in BDAccuri cytometer. Cells were gated by forward and side scatter for removing debris and doublets, and apoptotic cells were determined by Yo-Pro at FL-1 (ex 488; em 530±30 nm) and PI at FL-3 (ex 488; em 675LP nm) fluorescence.

rtPCR: Total RNA was isolated using RNAeasy Mini Kit (Quiagen) following the manufacturer’s instructions, and the concentration and purity of the RNA samples were determined using the Nanodrop Spectrophotometer 2000c. The cDNAs were synthesized by qScript cDNA Supermix (Quanta Biosciences) according to the protocol, followed by a quantitative polymerase chain reaction performed on the StepOnePlus Real-Time PCR System (Applied Biosystems). The thermal cycling conditions were composed of an initial de-naturalisation at 95°C for 10 min, followed by 40 cycles at 95°C for 15 sec, 60°C for 30 sec and 72°C for 30 sec. The amplifications were done using the SYBR Select Master Mix (Applied Biosystems).

The expression of TP53 was normalized against beta-actin with the following primers (TP53 Fw 5’-CCGCAGTCAGATCCTAGCG-3’; TP53 Rv 5’- AATCATCCATTGCTTGGGACG-3’; beta-actin Fw 5’-CATGTACGTTGCTATCCAGGC-3’; beta-actin Rv 5’-CTCCTTAATGTCACGCACGAT-3’).

Apoptosis Assay: Apoptosis was quantified using Hoechst staining to visualise apoptotic nuclei. Immunofluorescence staining for Flag allowed for visualization and quantification of Flag-positive cells via microscopy. 72 hours after transient transfection with Flag-PHD3 or Flag empty vector, U87 cells were incubated with Hoechst for 30 mins in incubator (37°C, 5% CO2). Cells were then fixed by 4% paraformaldehyde (PFA) for 15 mins and permeabilized by 0.1% Triton (0,1%) for 15 mins. Anti-Flag (mouse, 1:1000) was added to cells for overnight at 4°C. Secondary anti-mouse (Invitrogen, 1:1000) was added for 1 hour at room temperature. Approximately 200 Flag-positive cells were counted.

Crystal violet staining: U87 stable cell lines were transiently infected with adenovirus for PHD3 overexpression. Cells were then fixed and stained by crystal violet solution (0.1% crystal violet, 20% methanol, 80% dH2O) for 30mins at room temperature and washed 4 times with PBS.

Nuclear Cytoplasmic Fractionation: Cells were lysed in 40 mM HEPES, pH 7.5, 5 mMEGTA, 0.1% Nonidet P-40, 5 mM MgCl2 1 mM DTT, 1 mM VO4, 1 mM benzinamide. The lysate was centrifuged (800g, 4 minutes) to obtain the cytoplasmic fraction as supernatant. Nuclei were resuspended in 50 mM β-glycerophosphate, pH 7.3, 0.2 mMEDTA, 420 mM NaCl, 1.5 mM MgCl2 1 mM DTT, 25% glycerol, sonicated briefly on ice, vortexed, and centrifuged (18000g, 10 minutes); and the precipitated cell debris was discarded.

Halo-TUBE pulldown: Cells were treated with either 2 mM DMOG, 10 μM MG132 or DMSO for 2 hours. After the treatments cells were washed in ice-cold PBS and lysed in 50 mM Tris·HCl, pH 7.5, 1 mM EGTA, 1 mM EDTA, 1% (vol/vol) Triton X-100, 0.27 M sucrose, 10 mM sodium 2-glycerophosphate, 1 mM sodium ortho-vanadate, 50 mM NaF, 5 mM sodium pyrophosphate, protease inhibitor cocktail and 10mM Iodoacetamide. Lysates were cleared by centrifugation, 0.1 vol was separated to be loaded as the total lysate, and the rest were incubated with TUBE beads rocking at 4°C for 16 h. Beads were collected and washed three times with ice cold lysis buffer +500mM NaCl. Pellets were resuspended in 5× Laemmli buffer, boiled for 5 min at 55ºC, and fractionated by SDS-PAGE.

Supplemental References:

Turriziani, B., Garcia-Munoz, A., Pilkington, R., Raso, C., Kolch, W., and von Kriegsheim, A. (2014). On-beads digestion in conjunction with data-dependent mass spectrometry: a shortcut to quantitative and dynamic interaction proteomics. Biology 3, 320-332.
